# Medicinal plants used by women in Mecca: urban, Muslim and gendered knowledge

**DOI:** 10.1186/s13002-017-0193-4

**Published:** 2017-11-17

**Authors:** Afnan Alqethami, Julie A. Hawkins, Irene Teixidor-Toneu

**Affiliations:** 1 Section of Ecology and Evolutionary Biology (EEB), Harborne Building, School of Biological Sciences, University of Reading, Whiteknights, Reading, RG6 6AS UK; 2 Department of Biology, Faculty of Applied Science, Umm Al-Qura University, PO Box 715, Mecca, 21955 Saudi Arabia

**Keywords:** Ethnobotany, Saudi Arabia, Islam, Transmission, Food-medicine continuum

## Abstract

**Background:**

This study explores medicinal plant knowledge and use among Muslim women in the city of Mecca, Saudi Arabia. Ethnobotanical research in the region has focused on rural populations and male herbal healers in cities, and based on these few studies, it is suggested that medicinal plant knowledge may be eroding. Here, we document lay, female knowledge of medicinal plants in an urban centre, interpreting findings in the light of the growing field of urban ethnobotany and gendered knowledge and in an Islamic context.

**Methods:**

Free-listing, structured and semi-structured interviews were used to document the extent of medicinal plant knowledge among 32 Meccan women. Vernacular names, modes of preparation and application, intended therapeutic use and emic toxicological remarks were recorded. Women were asked where they learnt about medicinal plants and if and when they preferred using medicinal plants over biomedical resources. Prior informed consent was always obtained. We compared the list of medicinal plants used by these Meccan women with medicinal plants previously documented in published literature.

**Results:**

One hundred eighteen vernacular names were collected, corresponding to approximately 110 plants, including one algae. Of these, 95 were identified at the species level and 39 (41%) had not been previously cited in Saudi Arabian medicinal plant literature. Almost one half of the plants cited are food and flavouring plants. Meccan women interviewed learn about medicinal plants from their social network, mass media and written sources, and combine biomedical and medicinal plant health care. However, younger women more often prefer biomedical resources and learn from written sources and mass media.

**Conclusions:**

The fairly small number of interviews conducted in this study was sufficient to reveal the singular body of medicinal plant knowledge held by women in Mecca and applied to treat common ailments. Plant availability in local shops and markets and inclusion in religious texts seem to shape the botanical diversity used by the Meccan women interviewed, and the use of foods and spices medicinally could be a global feature of urban ethnobotany. Ethnobotanical knowledge among women in Islamic communities may be changing due to access to mass media and biomedicine. We recognise the lack of documentation of the diversity of medicinal plant knowledge in the Arabian Peninsula and an opportunity to better understand gendered urban and rural knowledge.

## Background

### The use of medicinal plants in urban environments

Currently, more people live in cities than in rural areas, and urban populations continue to grow: by 2050, two thirds of the world’s population will live in cities [1]. Urbanisation brings new health challenges resulting from ease of contagion, maintenance of disease due to high population densities and stress-related ailments [2]. Although biomedicine is often easily available in urban settings, traditional medicines can still be the most convenient and affordable health care resource [2, 3]. Similar to people in rural areas, urban dwellers can hold rich medicinal plant knowledge. Recent studies evidence the dynamism and adaptive nature of urban medicinal plant knowledge (e.g. [3–7]), challenging views that such knowledge is lost in cities.

Since Balick et al. [4] noted a lack of ethnobotanical studies in urban areas, urban ethnobotany has flourished. Ethnobotanical surveys in cities have focused on issues such as the change of plant use by international immigrants [4, 6, 8–12] or the ethnobotanical diversity found in urban and peri-urban markets [13–15] and home gardens [16, 17]. With few exceptions [7, 14, 15], these studies are set in Northern and Western countries. Cities in other parts of the world are equally dynamic plant knowledge hubs, and some have played an important role in the trade of medicinal plant material and knowledge historically. This is the case of cities along the silk and incense trade routes in the Middle East [18–20].

### Women’s medicinal plant knowledge

Several studies across the world have reported the pivotal role of women as holders of medicinal plant knowledge [21–28]. Medicinal plant knowledge is often women’s cultural domain because of the role women play in providing household care [21–23, 29, 30]. It also stems from gendered labour and spaces, which affect plant resource harvesting and management [21, 22, 26]. Women are often more knowledgeable about medicinal plant identification and use than are men [21, 23–25], and their knowledge can be epistemologically different [22].

Moreover, the documentation of traditional knowledge as part of plant biodiversity research has historically been gender-biased towards men, which can result in misleading and incomplete results [22, 31, 32]. Both Howard [22] and Pfeiffer and Butz [32] claim that focus on male specialists, such as shamans and herbalists, has ignored a wealth of lay, female plant knowledge in ethnobotanical research. Historically, ethnobotanists have been predominantly male, which hampered their access to women’s knowledge in societies where men are granted greater public access than women [32]. These considerations are particularly important in conducting ethnobotanical research in the Islamic world, where gendered spaces and networks are particularly strong. Women in urban Islamic contexts have so far gone unnoticed by ethnobotanical enquiry, although they are the main medicinal plant users in Saudi Arabian cities [33].

### Islam and medicinal plants

In the Arabian Peninsula, where this study took place, the use of local plant diversity for medicines is part of the cultural heritage [34, 35] and is currently embedded in Islamic medicinal practices. Islamic medicine integrates ancient Greek medicine, which first arrived to the Islamic world through translations of the works of Hippocrates, Dioscorides and Galen [36], with the teachings of the Prophet Mohamed (Hadith) referring to health, disease and medical treatment that became known as ‘The Medicine of the Prophet’ [37, 38]. From ancient Greek medicine, understandings of the functioning of the body through the humoral system and the view of disease as a loss of balance became part of Islamic medicine [37, 39, 40]. Arabian physicians, such as Al Razi, Ibn Sina, Aby Al Kassin Al Zahrawi, Ibn Rushd and Ibn Naffs, further developed medicine as a scientific discipline in the Middle Ages [36].

Islamic religious practices generate specific cultural behaviours that aim to preserve health, and early Islamic medical tradition focused on preventive rather than therapeutic medicine [37–39]. Beliefs in jinni and Evil Eye as causes of illness are also common in the Arab world [29, 36, 40]. These magical features are possibly elements of the Bedouin world view that became legitimised by the Quran and the Hadith [40]. Stemming from religious teachings, Islamic medicine has a holistic view of health, where physical, spiritual, psychological, social and environmental factors are intertwined [38, 39]. The maintenance of health and recovery from illness are both a physical and spiritual process, underpinned by the belief in God [38, 39].

### Study aims

Whilst medicinal plant uses are under-documented in the Middle East [41, 42] and a trend of loss of ethnobotanical and ethnomedicinal knowledge is observed in this area [36, 43], the extent of medicinal plant knowledge held by urban women remains unknown. The aim of this study was to document urban women’s medicinal plant knowledge in an Islamic context, identifying the plant species used. Additionally, modes of transmission of knowledge were evaluated, evidence for change noted. The extent to which women’s medicinal plant knowledge has been under-documented was inferred by comparing results from free-listing and semi-structured interviews conducted with women in Mecca to selected published literature on medicinal plants used in Saudi Arabia.

## Methods

### Research setting: Mecca as a study site

**Fig. 1:**
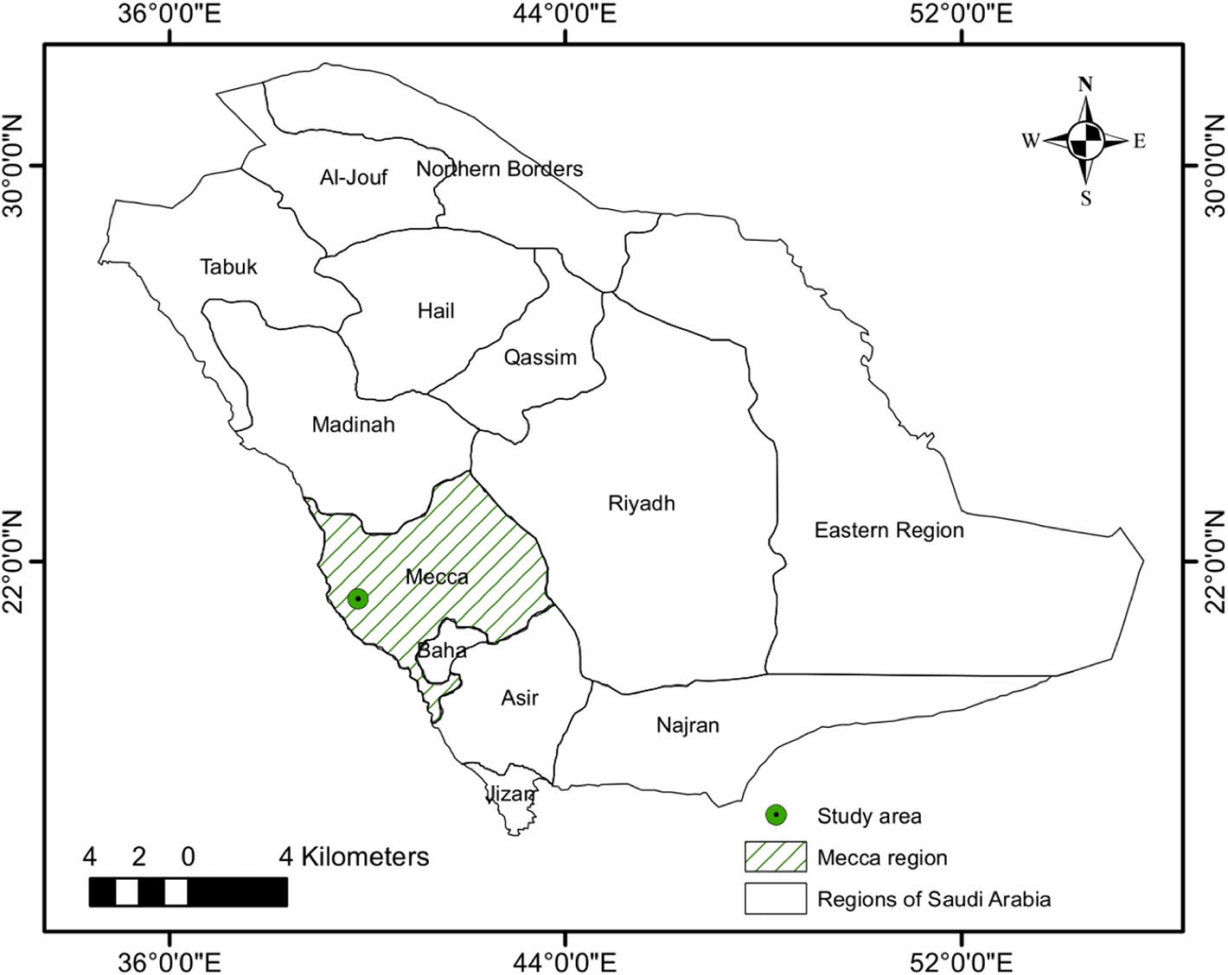
Map of Saudi Arabia with regional divisions. The city of Mecca is indicated by a dot

The city of Mecca (Kingdom of Saudi Arabia) is located in a narrow valley 80 km south of Jeddah on the Red Sea coast, west of the Arabian Peninsula (Fig. 1). The city is the capital of the Mecca region, neighbouring the regions of Medina in the north, Baha and Asir in the south and Al-Riyad in the east. The region of Mecca is situated in a subtropical dry environment [44]; its vegetation is dominated by xerophytic species and composed of floristic elements from the Saharo-Arabian, Irano-Turanian and Sudano-Zambian biogeographical regions [45].

Mecca was the birthplace of the Prophet Muhammad and the site of the first Quranic revelation. It is regarded as the most holy city in Islam and the pilgrimage to Mecca, known as Hajj, is obligatory for all able Muslims. Due to the high numbers of pilgrims travelling to Mecca, the city is the most culturally diverse in the Islamic world. Saudi Arabians may use both traditional herbal and biomedical treatments [34, 36, 45], and a wide range of health care resources are available in Mecca: from traditional medicines and healers ([45], Al-Qethami, pers. obs), to modern biomedical care provided by the Saudi government for free to all Muslims.

### Conducting interviews with Meccan women

The ethical guidelines of the Code of Ethics of the International Society of Ethnobiology [46], the Declaration of Helsinki [47] and University of Reading ethical protocols were followed in this research. Approval from the Ethics Committee of the School of Biological Sciences, University of Reading, was obtained (Research Ethics Project Submission SBS15-16 11).

Meccan women were interviewed by the first author from May to June 2016; individual free-listing and semi-structured interviews were conducted with 32 female adults. Targeted sampling was used for selecting informants who use medicinal plants [48] from the first author’s social network. Snowball sampling was later used to identify other local women who use medicinal plants [49]. All the informants were born in the Mecca region, with ages ranging between 28 and 69 years old (see Table 1 for the informants’ anonymous social data). Although older informants may hold further knowledge, interviews were conducted until a saturation of information was reached. Prior informed consent was obtained verbally from each woman before they were interviewed. Interviews were conducted in Arabic and recorded when the agreement from the informants was obtained (*n* = 9). Firstly, women were asked to list the medicinal plants they knew, documenting plant names. Then, semi-structured interviews were used to elucidate the parts used, therapeutic uses, preparation and administration processes (including use in mixtures) and perceptions of potential toxicity and side effects of plants used. Moreover, women were asked about how they had acquired this knowledge, if medicinal plants or biomedicine were preferred and when medicinal plants where preferred over biomedicine. The resulting ethnographic data were useful to understand women’s attitudes, beliefs and therapeutic goals underpinning medicinal plant use.

**Table 1:** Informant’s details. Place of residence is at the time of the interview; three women were temporarily living in the UK, where they were interviewed. MP/BM refers to preference for medicinal plants or biomedicine

Code	Age	Occupation	Literacy	*N* of children	Place of residence	*N* of household members	Place of origin	Number of plants listed	MP/BM	Source knowledge
inf1	30	Lecturer	PhD (Arabic, English)	1	Reading, UK	3	Mecca	15	BM	Scientific lectures, grandparents and mother
inf2	47	Lecturer	PhD (Arabic, English)	6	Reading, UK	8	Jeddah	13	MP	Grandmothers and mother
inf3	35	Housewife	Secondary education (Arabic)	5	Mecca	7	Mecca	9	BM	Grandmothers and mother
inf4	31	Student	PhD (Arabic, English)	0	Reading, UK	9	Mecca	10	BM	Mother, scientific lectures, Internet
inf5	49	Housewife	Primary education (Arabic)	6	Mecca	8	Mecca	23	MP	Mother
inf6	51	Housewife	Primary education (Arabic)	3	Mecca	4	Mecca	18	MP	Grandmothers
inf7	32	Housewife	Secondary education (Arabic)	5	Mecca	7	Mecca	21	MP	Grandmothers, mother, neighbours, books, Internet, television
inf8	49	Housewife	Primary education (Arabic)	5	Mecca	6	Mecca	13	MP	Grandmothers
inf9	50	Housewife	Primary education (Arabic)	4	Mecca	5	Mecca	24	MP	Written sources, mother and aunt
inf10	60	Housewife	Primary education (Arabic)	10	Mecca	5	Mecca	18	MP	Grandmothers
inf11	45	Housewife	Primary education (Arabic)	2	Mecca	3	Mecca	24	MP	Grandmothers
inf12	56	Housewife	Primary education (Arabic)	2	Mecca	4	Mecca	24	MP	Grandmothers
inf13	32	Housewife	Bachelor (Arabic)	4	Mecca	6	Mecca	9	BM	Mother
inf14	65	Housewife	Illiterate (Arabic)	9	Mecca	10	Mecca	12	MP	Grandmothers
inf15	34	Housewife	Secondary education (Arabic)	2	Mecca	4	Mecca	12	BM	Wider community (social network beyond family members)
inf16	29	Housewife	Bachelor (Arabic)	3	Mecca	5	Mecca	15	BM	Mother, Internet
inf17	69	Housewife	Illiterate (Arabic)	7	Mecca	9	Mecca	17	MP	Grandmothers
inf18	60	Housewife	Illiterate (Arabic)	9	Mecca	6	Mecca	25	MP	Mother, Internet, shops of medicinal plants
inf19	30	Housewife	Bachelor (Arabic)	3	Mecca	5	Mecca	8	BM	Mother, Internet
inf20	59	Housewife	Primary education (Arabic)	3	Mecca	4	Mecca	18	MP	Grandmothers
inf21	50	Housewife	Illiterate (Arabic)	6	Mecca	8	Mecca	17	MP	Grandmothers
inf22	45	Housewife	Bachelor (Arabic)	6	Mecca	8	Mecca	16	MP	Grandmothers
inf23	37	Housewife	Secondary education (Arabic)	5	Mecca	7	Taif	12	MP	Grandmothers
inf24	39	Housewife	Bachelor (Arabic)	4	Mecca	6	Mecca	17	MP	Grandmothers
inf25	34	Housewife	Bachelor (Arabic)	3	Mecca	5	Mecca	12	BM	Mother
inf26	33	Housewife	Bachelor (Arabic)	3	Mecca	5	Mecca	12	BM	Family
inf27	33	Housewife	Bachelor (Arabic)	4	Mecca	6	Mecca	24	MP	Family, nutrition studies at the university
inf28	34	Housewife	Bachelor (Arabic)	6	Mecca	8	Mecca	17	BM	Mother, family, friends, Internet
inf29	28	Lecturer	Bachelor (Arabic)	1	Mecca	3	Mecca	18	BM	Mother, sisters, school
inf30	46	Housewife	Bachelor (Arabic)	4	Mecca	6	Mecca	17	MP	Mother, family, books
inf31	60	Housewife	Primary education (Arabic)	9	Mecca	5	Mecca	12	MP	Grandmothers
inf32	60	Housewife	Illiterate (Arabic)	10	Mecca	5	Mecca	10	MP	Grandmothers

### Analysis of medicinal plant’s salience

Data collected during interviews were structured in ‘use reports’. A ‘use report’ is one citation of one plant use by one informant, and it includes vernacular name, part used, local use, preparation and administration. Alongside the emic use citations, therapeutic applications were classified by disease categories according to the International Classification of Primary Care, as recommended by Staub et al. [50]. The software *anthropac* [51] was used to analyse free-lists, obtaining the frequency of citation and Smith’s index for each plant [52]. Smith’s index is a measure of the cultural importance of each plant depending on the frequency of citation and its rank in the free-lists [52].

### Plant collection and identification

**Fig. 2:**
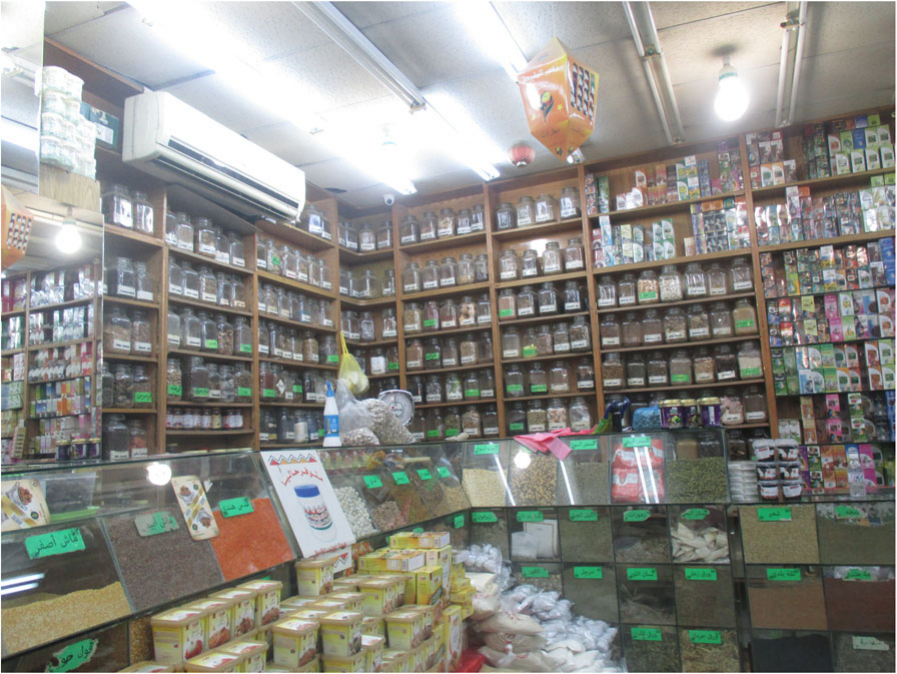
Herbalist store in Mecca. *Atar AlKuwait*, one of the most famous establishments selling medicinal plant products in Mecca

Most voucher specimens were obtained directly from informants. When this was not possible, they were obtained from local shops and supermarkets (*Atar AlKuwait*, *Atar Alamana* and *Matager Alsudia*; Fig. 2). Voucher specimens were not obtained for two plants; specimens from the Umm Al-Qura University herbarium were used to identify these according to vernacular names. Voucher specimens (including market samples) were deposited in the Umm Al-Qura University herbarium. Since no plants were collected from the wild directly, collection permits were not necessary. Plant identification was carried out by the first author in the herbarium of Umm Al-Qura University using the *Flora of Saudi Arabia* [53], and identifications were validated by a plant taxonomist in Umm Al-Qura University. Nomenclature and family adscriptions follow The Plant List [54], and the list was contrasted with the online checklist of the *Flora of Saudi Arabia* [55].

### Literature review

In order to assess the documented Saudi Arabian medicinal plant knowledge, a systematic literature review was conducted. Google Scholar, the Saudi Digital Library, Research Gate and King Abdullah bin Abdulaziz Library were searched for books and scientific articles using the search terms ‘Saudi Arabia’, ‘Mecca’, ‘Medicinal Plant’, ‘Herbal’, ‘Traditional Medicine’ or ‘Ethnobotany’ in English and Arabic, with no restriction on date of publication. Literature were excluded if they only reported pharmacological activities without mention of local knowledge sources, or if they focused on veterinary plant uses. Eleven sources were identified, obtained and reviewed, including seven journal articles and four books. For these, the methods were reviewed, examining how local names and plant uses were recorded and how plant identification was achieved. Explicit knowledge documentation methods, collection of herbarium specimens, the literature used to identify them and deposition in herbaria were quality criteria used to identify rigorous literature sources [5, 56]. One book and three scientific articles were considered rigorous and selected to compare the medicinal plants listed with those used by the women in Mecca interviewed during this study. The names from selected literature were cross-checked with The Plant List [54] to identify accepted plant names and family assignments. Botanical species, vernacular names and uses mentioned by women in Mecca during the field study were then compared to those in the selected literature to identify the extent of the overlap between lists.

## Results

### Medicinal plants used by women in Mecca

In total, 753 use reports were collected during interviews and 118 medicinal plant vernacular names were documented, belonging to approximately 110 botanical taxa (43 families; Table 2), including one algae (*Fucus vesiculosus*). Ninety-five medicinal plants were identified at the species level, 12 were identified at the genus level, one at the family level and two could not be identified. The most common medicinal families are Apiaceae (10%; 11 taxa), Fabaceae (9%; 10 taxa) and Lamiaceae (7%; eight taxa). Asteraceae, Brassicaceae and Poaceae were represented by five taxa each; Myrtaceae, Rosaceae and Zingiberaceae by four taxa each; and Amaranthaceae, Apocynaceae, Burseraceae and Rutaceae by three taxa each. Seven families were represented by two taxa and 24 families were represented only by one taxon. The most cited salient medicinal plants during the interviews are *helba* (*Trigonella foenum-graecum*), *kamun* (*Cuminum cyminum*), *yansun* (*Pimpinella anisum*), *qurfa* (*Cinnamomum verum*) and *zanajabil* (*Zingiber officinale*) (Table 2). Interestingly, we observed that one third (32%) of the plants mentioned in the interviews are common vegetable and fruit crops, and almost one fifth (17%) are spices. More than half of the taxa were not native to Saudi Arabia (54%; Table 2).

**Table 2:** Comprehensive inventory of the plants listed by women in Mecca including the scientific name and family, whether the plant is found in the Flora of Saudi Arabia and whether it is used as a food or spice, vernacular name(s), part(s) used, therapeutic use categories, preparation, administration, toxicity and side effects, frequency of citation and Smith’s S. For presence or absence in the Flora of Saudi Arabia, Y = yes, N = no, and for food and/or spice use, F = food and S = spice. Plants not documented in the selected literature are marked with ‘*’

Scientific name (family, voucher)	Flora of Saudi Arabia	Food (F) or spice (S)	Vernacular name(s) (Arabic)	Part(s) used	Therapeutic use categories	Preparation	Administration	Toxicological remarks and reported side effects	Frequency of citation	Smith’s S
*Acacia nilotica* (L.) Delile. (Fabaceae, EWM_62)	N	–	Qard (ضرق)	Fruit, root	Neurological, digestive	Infusion, decoction	Wash, bath	N	2	0.041
*Acacia senegal* (L.) Willd. (Fabaceae, EWM_77)	N	–	Samg arabi (يبرع غمص)	Resin	Urological, endocrine and nutritional	Infusion	Oral ingestion (drink)	N	2	0.042
*Aerva javanica* (Burm.f.) Juss. ex Schult. (Amaranthaceae, EWM_90)	Y	–	Tarf (فرط)	All plant	Neurological, digestive	Infusion, ground	Oral ingestion (drink), put directly on the teeth	N	1	0.009
* *Alchemilla* sp. (Rosaceae, EWM_68)	Y	–	Rajel alasad (دسلآا لجر)	Root	Digestive	Infusion	Oral ingestion (drink)	Not to be used by pregnant women	1	0.01
*Alchemilla xanthochlora Rothm. (Rosaceae, EWM_01)	N	–	Abat alseda (ةأبع ةديسلا)	Leaf	Gynaecological	Infusion	Oral ingestion (drink)	N	1	0.021
*Allium cepa* L. (Amaryllidaceae, EWM_14)	N	F	Bsal (لصب)	Bulb, tunic	General and unspecified, gynaecological	Juice with lemon, added to food	Oral ingestion (drink or eat), fumigation, placing onions on the bottom of the feet and wear socks	N	4	0.034
*Allium sativum* L. (Amaryllidaceae, NA)	N	F	Thoom (موث)	Bulb	General and unspecified, cardiovascular, digestive, ear	Put with food, in water, mash, no preparation	Oral ingestion (eat or drink), put directly on the teeth	N	7	0.069
*Aloe* sp. (Xanthorrhoeaceae, EWM_74)	Y	–	Sabr (ربص)	Leaf flesh	Digestive, gynaecological, skin	Put in water	Oral ingestion (drink), topic on wounds	Causes diarrhoea	1	0.007
*Aloe vera* (L.) Burm.f. (Xanthorrhoeaceae, EWM_73)	N	–	Sabbar (رابص)	Leaf flesh	Skin	No preparation	Topic on wounds or hair	Overdose may cause colon cancer	2	0.041
*Alpinia officinarum Hance (Zingiberaceae, EWM_43)	N	S	Kholanjan (ناجنلوخ)	Rhizome	Digestive, cardiovascular, skin	Infusion	Oral ingestion (drink)	N	1	0.013
*Ammi visnaga (L.) Lam. (Apiaceae, EWM_44)	Y	S	Khull (ةلخ)	Seed, leaf, fruit	Cardiovascular, urological	Infusion	Oral ingestion (drink)	Overdose may cause dryness and hypotension, not to be used by pregnant women	4	0.053
*Anastatica hierochuntica* L. (Brassicaceae, EWM_34)	Y	–	Kaff maryam (فك ميرم)	Fruit	Family planning, gynaecological	Infusion	Oral ingestion (drink), topic	Can cause stomach disorders and nausea	4	0.04
*Anethum graveolens* L. (Apiaceae, EWM_81)	Y	S	Shabath (ثبش)	Leaf	Digestive	Infusion	Oral ingestion (drink)	Not to be used by those who suffer from kidney disease	1	0.004
*Apium graveolens L. (Apiaceae, EWM_46)	Y	F	Korfos (سفرك)	Leaf	Gynaecological, digestive, neurological	Infusion, juice, no preparation	Oral ingestion (eat or drink)	Not to be used by pregnant women	2	0.02
*Artemisia judaica L. (Asteraceae, EWM_87)	Y	–	Shayh (حيش)	All plant	Digestive	Infusion	Oral ingestion (drink)	Not to be used by pregnant women	1	0.007
*Avena sativa L. (Poaceae, EWM_88)	Y	F	Shuran (نافوش)	Seed	Endocrine and nutritional	Ground	Oral ingestion (eat)	N	1	0.002
*Azadirachta indica* A. Juss. (Meliaceae, EWM_60)	N	–	Nim (مين)	Leaf	Digestive, general and unspecified	Decoction	Oral ingestion (drink), bath	N	2	0.035
*Beta vulgaris L. (Amaranthaceae, EWM_09)	Y	F	Banjr (رجنب)	Fruit	Blood and immune mechanisms	Decoction, juice, no preparation	Oral ingestion (drink ore at)	N	7	0.054
*Boswellia sacra* Flueck. (Burseraceae, EWM_50)	N	–	Laban aldakar, laban shahari (نابل ،ركذلا يرحش نابل)	Resin	General and unspecified, respiratory, neurological	Infusion, chewing, fumigation	Oral ingestion (drink)	N	8	0.113
*Brassica oleracea L. (Brassicaceae, NA)	N	F	Kronb (بنرك)	Leaf	Digestive	Juice (with apples and milk), in food, decoction	Oral ingestion (drink or eat)	N	7	0.018
*Brassica rapa* L. (Brassicaceae, EWM_40)	N	F	Khardal (لدرخ)	Seed	Musculoskeletal, cardiovascular	Ground, infusion	In hot water (to put feet in)	N	2	0.028
*Calotropis procera* (Ait.) Ait. fil., (Apocynaceae, NA)	Y	–	Eshr (راشع)	Flower	Respiratory	In water, ground	Oral ingestion (drink or eat)	Can cause diarrhoea	1	0.005
*Camellia sinensis (L.) Kuntze (Theaceae, EWM_85)	N	F	Shay, shay akhdar (ياش, رضخأ ياش)	Leaf	Digestive, metabolic and nutritional, gynaecological, general and unspecified, family planning	Decoction, infusion	Topic, oral ingestion (drink), fumigation	When ingested, overdose may cause diarrhoea	7	0.09
*Carthamus tinctorius* L. (Asteraceae, EWM_61)	Y	–	Osforr (رفصع)	Flower	Endocrine and nutritional, cardiovascular	Infusion	Oral ingestion (drink)	Overdose may cause diarrhoea	1	0.03
*Carum carvi L. (Apiaceae, EWM_37)	N	S	Karawia (ةيوارك)	Seed	Digestive, gynaecological, general and unspecified, respiratory, family planning	Decoction	Oral ingestion (drink)	Overdose may affect the kidneys	6	0.111
*Ceratonia siliqua* L. (Fabaceae, EWM_41)	N	F	Kharnub (بونرخ)	Fruit	Digestive	Infusion	Oral ingestion (drink)	N	1	0.025
*Cinnamomum verum J. S. Presl (Lauraceae, EWM_67)	N	S	Qurfa (هفرق)	Bark	Gynaecological, digestive, blood and immune system, endocrine and nutritional, respiratory, family planning	Decoction, ground	Oral ingestion (eat or drink)	Overdose may cause fainting, hypotension, dizziness and sweating, bleeding if used for more than 3 days, not to be used with ginger, not to be used by pregnant women (may cause abortion)	20	0.349
*Citrullus colocynthis* (L.) Schrad. (Cucurbitaceae, EWM_28)	Y	–	Hanzal (لظنح)	Leaf, fruit	Digestive, skin	Infusion, no preparation	Oral ingestion (drink), topic	N	2	0.041
*Citrus sinensis (L.) Osbeck (Rutaceae, EWM_51)	N	F	Portokal (لاقترب)	Fruit	Digestive, general and unspecified	Juice, no preparation	Oral ingestion (eat or drink)	N	2	0.04
*Citrus* sp. (Rutaceae, EWM_51)	N	F	Limon (نوميل)	Fruit	General and unspecified, digestive, endocrine and nutritional, respiratory	Juice, dry until black and ground in water, decoction	Oral ingestion (drink), topic	N	10	0.201
*Coffea arabica* L. (Rubiaceae, EWM_64)	N	F	Qashr album (رشق نبللا)	Pericarp	Blood and immune system, gynaecological, endocrine and nutritional	Decoction, infusion	Oral ingestion (drink)	Not to be used by pregnant women	6	0.117
*Commiphora gileadensis* (L.) C. Christ. (Burseraceae, EWM_12	Y	–	Basham (ماشب)	Root	Gynaecological, general and unspecified, respiratory, neurological	Decoction, infusion	Oral ingestion (drink), topic	N	3	0.051
*Commiphora myrrha* (Nees) Engl. (Burseraceae, EWM_53)	Y	–	Marr (رم)	Resin	Skin, digestive, respiratory, gynaecological, general and unspecified	In water, infusion	Poultice, mouthwash, oral ingestion (drink)	Overdose may upset the stomach and cause general discomfort	17	0.323
*Coriandrum sativum* L. (Apiaceae, EWM_47)	Y	F	Kozbra (ةربزك)	Leaf	Digestive, cardiovascular, neurological	Ground, infusion, with food	Oral ingestion (eat or drink)	Overdose may cause infertility	5	0.079
*Costus sp. (Costaceae, EWM_63)	N	–	Qasd hindi (يدنه دصق)	Root	Endocrine and nutritional, general and unspecified, gynaecological, blood and immune system, urological, family planning	Ground, decoction	Oral ingestion (eat or drink)	N	4	0.05
*Crocus sativus L. (Iridaceae, EWM_99)	N	S	Zafran (نارفعز)	Stigma	General and unspecified, digestive, respiratory	With food, in water	Oral ingestion (eat or drink)	Not to be used by pregnant women	3	0.025
*Cucumis sativus* L. (Cucurbitaceae, NA)	N	F	Khiar (رايخ)	Fruit	Urological, digestive, neurological	No preparation	Oral ingestion (eat)	N	2	0.033
*Cuminum cyminum* L. (Apiaceae, EWM_36)	Y	S	Kamun (نومك)	Seed	Digestive, gynaecological, endocrine and nutritional, general and unspecified, respiratory	Infusion, decoction, ground	Oral ingestion (eat or drink)	Overdose may upset the stomach and produce constipation)	22	0.486
*Curcuma longa* L. (Zingiberaceae, EWM_38)	N	S	Karrakum (مكرك)	Rhizome	Musculoskeletal, skin, blood and immune system, general and unspecified, digestive, respiratory, endocrine and nutritional	Ground, infusion, with honey	Oral ingestion (eat or drink), poultice	Overdose may upset the stomach	8	0.09
*Cydonia oblonga Mill. (Rosaceae, NA)	N	F	Safarjil (لجرفس)	Fruit	Digestive	No preparation	Oral ingestion (eat)	N	1	0.02
*Cymbopogon schoenanthus (L.) Spreng. (Poaceae, EWM_02)	Y	–	Adhkhur (رخذا)	Leaf	Neurological, digestive, urological, general and unspecified	Decoction	Oral ingestion (drink), decoction (steam inhalation)	N	3	0.069
*Dialium sp. (Fabaceae, EWM_26)	N	–	Hamayd (ضيمح)	Fruit	Digestive, urological	Infusion	Oral ingestion (drink)	N	1	0.008
*Dipterygium glaucum Decne. (Cleomaceae, EWM_06)	Y	–	Arfaj (جفرع)	Leaf	Digestive, respiratory	Infusion	Oral ingestion (drink), decoction (steam inhalation)	N	1	0.003
*Dracaena cinnabari Balf.f. (Asparagaceae, EWM_16)	N	–	Dam alakhwan (مد نيوخلأا)	Resin	General and unspecified, digestive	Ground	Poultice	N	1	0.006
*Elettaria cardamomum (L.) Maton (Zingiberaceae, EWM_24)	N	S	Hal (لاه)	Fruit, seed	Respiratory	Infusion	Oral ingestion (drink)	N	1	0.004
*Eruca* sp. (Brassicaceae, EWM_33)	Y	–	Jarjir (ريجرج)	Leaf	Blood and immune system, respiratory	With food, no preparation	Oral ingestion (eat or drink)	N	9	0.133
*Eucalyptus camaldulensis* Dehnh. (Myrtaceae, EWM_35)	N	–	Kafor (روفاك)	Leaf	Neurological, digestive, general and unspecified	Infusion, decoction	Oral ingestion (drink)	N	3	0.035
*Ferula assa-foetida* L. (Apiaceae, EWM_25)	N	–	Halatayta (ةتيتلح)	Resin	Digestive, respiratory	Maceration	Oral ingestion (drink)	Not to be used by breastfeeding women, children should not take more than 5 ml	4	0.068
*Ficus palmata* Forssk. (Moraceae, NA)	Y	F	Hamat (طامح)	Fruit	Digestive	No preparation	Oral ingestion (eat)	N	1	0.007
*Foeniculum vulgare* Mill. (Apiaceae, EWM_83)	Y	S	Shamr (رمش)	Seed	Digestive, urological, gynaecological, general and unspecified, neurological, blood and immune system	Infusion, decoction, ground	Oral ingestion (drink or eat)	Overdose may cause inflammation of the intestines	14	0.248
*Fucus vesiculosus L. (Fucaceae, EWM_21)	N	–	Foqus (سقوف)	Leaf	Endocrine and nutritional, cardiovascular	Infusion	Oral ingestion (drink)	N	1	0.004
*Glebionis coronaria (L.) N.N. Tzvel. (Asteraceae, EWM_04)	N	–	Aqhwan (ناوحقا)	Leaf, flower	Digestive	Infusion	Oral ingestion (drink)	Not recommended for babies	1	0.004
*Glycyrrhiza glabra* L. (Fabaceae, EWM_18)	Y	–	Eirqsos (سوسقرع)	Root	Digestive, musculoskeletal	Infusion	Oral ingestion (drink)	N	1	0.023
*Hibiscus sabdariffa L. (Malvaceae, EWM_49)	N	–	Kwajara karakadi (،يتارجوك ةيدكرك)	Calyx, sepal	Cardiovascular, blood and immune system, digestive, urological, neurological	Infusion, decoction	Oral ingestion (drink)	N	15	0.209
*Hordeum vulgare L. (Poaceae, EWM_82)	Y	F	Shaeir (ريعش)	Seeds	Urological, digestive	Decoction	Oral ingestion (drink)	Not to be used by pregnant women	5	0.041
*Hyphaene sp. (Arecaceae, EWM_17)	Y	–	Dom (مود)	Fruit	Cardiovascular	Ground, infusion with honey	Oral ingestion (drink)	N	1	0.026
*Juniperus procera* Hochst. ex Endl. (Cupressaceae, EWM_05)	Y	–	Arar (رعرع)	Fruit	Urological	Infusion, decoction	Oral ingestion (drink)	Not to be used by pregnant women	1	0.006
*Lactuca sativa L. (Asteraceae, NA)	N	F	Khas (سخ)	Leaf	Blood and immune system, urological, digestive	With food	Oral ingestion (eat)	N	2	0.02
*Laurus nobilis L. (Lauraceae, EWM_93)	N	S	Waraq alghar (قرو راغلا)	Leaf	Urological, general and unspecified	Infusion	Oral ingestion (drink)	N	1	0.007
*Lawsonia inermis* L. (Lythraceae, EWM_27)	Y	–	Hana (انح)	Leaf	Neurological, musculoskeletal, skin	Ground, with honey in water	Topic, poultice	N	3	0.03
*Lepidium sativum* L. (Brassicacea, EWM_70)	Y	S	Rashad, thafa (،داشر ءافث)	Seed	Gynaecological, musculoskeletal, skin, digestive, endocrine and nutritional, neurological, general and unspecified, cardiovascular	Ground, infusion, no preparation	Oral ingestion (eat or drink), poultice	Not to be used for over a month, nor by pregnant women, overdose may cause diarrhoea and upset stomach	13	0.228
*Linum usitatissimum* L. (Linaceae, EWM_13)	Y	–	Bidharrat alkitan (ةرذب ناتكلا)	Seed	Digestive, endocrine and nutritional, general and unspecified, gynaecological	Ground, infusion	Oral ingestion (eat or drink)	Can cause stomach and abdominal pain, and gases	4	0.079
*Lupinus albus L. (Fabaceae, EWM_91)	N	F	Tarmas (سمرت)	Seed	Neurological, endocrine and nutritional, blood and immune system	With food, infusion, decoction	Oral ingestion (eat or drink)	Overdose may cause phlegm and skin yellowing	4	0.035
*Lycopersicon esculentum* Mill. (Solanaceae, NA)	N	F	Tamatum (مطامط)	Fruit	Blood and immune system	No preparation	Oral ingestion (eat)	Overdose may cause diarrhoea	1	0.023
*Malva parviflora* L. (Malvaceae, EWM_39)	Y	–	Khabiza (ةزيبخ)	Leaf, flower	Family planning, general and unspecified, respiratory	Infusion, no preparation	Chewing, oral ingestion (drink)	N	1	0.002
*Matricaria aurea* (L.) Sch. Bip. (Asteraceae, EWM_07)	Y	–	Babunj (جنوباب)	Flower	General and unspecified, neurological, cardiovascular, digestive, respiratory	Infusion	Inhale, oral ingestion (drink)	Overdose may cause dizziness or headache	9	0.134
*Melissa officinalis L. (Lamiaceae, EWM_52)	N	–	Malisaa (اسيلم)	Leaf	Digestive	Infusion	Oral ingestion (drink)	Not to be used by pregnant women or children	1	0.019
*Mentha* sp. (Lamiaceae, EWM_59)	Y	–	Nena (عانعن)	Leaf	Digestive, cardiovascular, general and unspecified, neurological, respiratory, gynaecological	Decoction, infusion	Oral ingestion (drink)	N	16	0.289
*Morus nigra* L. (Moraceae, EWM_95)	N	F	Waraq tawt (توت قرو)	Leaf	Family planning	Decoction	Oral ingestion (drink)	N	1	0.013
*Musa acuminata Colla (Musaceae, NA)	N	F	Moz (زوم)	Fruit	Digestive	No preparation	Oral ingestion (eat)	N	1	0.029
*Myrtus communis* L. (Myrtaceae, EWM_92)	Y	–	Waraq alas (سلاا قرو)	Leaf	General and unspecified, respiratory, blood and immune system, cardiovascular	Decoction, infusion	Oral ingestion (drink)	N	2	0.022
*Nigella sativa* L. (Ranunculaceae, EWM_22)	N	S	Haba sawda (ةبح ءادوس)	Seed	Blood and immune system, general and unspecified, respiratory, gynaecological, musculoskeletal, neurological	Ground, 7 seeds with honey or dates, no preparation	Oral ingestion (eat or drink)	Not to be used by pregnant women, children, or babies, if more than 7 seeds are taken in a day, it can cause abortion, overdose may affect the kidneys	13	0.201
*Ocimum basilicum* L. (Lamiaceae, EWM_71)	N	–	Rihan (ناحير)	Leaf, flower	General and unspecified, respiratory	Infusion	Oral ingestion (drink)	N	1	0.006
*Ocimum* sp. (Lamiaceae, EWM_23)	Y	–	Habaq (قبح)	Leaf	General and unspecified, digestive	Decoction, infusion	Oral ingestion (drink)	Not to be used by pregnant women or children	3	0.04
*Olea europaea* L. (Oleaceae, EWM_101)	Y	F	Zeetoun (نوتيز)	Oil, leaf	General and unspecified, endocrine and nutritional, respiratory	Syrup, liniment, infusion, decoction	Oral ingestion (eat or drink), liniment	If boiled (leaves) may become toxic, overdose may cause diarrhoea	5	0.086
*Origanum majorana L. (Lamiaceae, EWM_11)	N	–	Bardaqush (شوقدرب)	Leaf	Endocrine and nutritional, urological, digestive, musculoskeletal, cardiovascular	Infusion, decoction	Oral ingestion (drink)	N	5	0.078
*Pennisetum glaucum (L.) R.Br. (Poaceae, EWM_15)	Y	F	Dakhun (نخد)	Seed	Digestive	Ground	Oral ingestion (eat)	N	1	0.015
*Petroselinum crispum* (Mill.) Fuss (Apiaceae, EWM_10)	N	F	Baqdunas (سنودقب)	Leaf	Cardiovascular, urological, female, gynaecological, blood and immune system, musculoskeletal, skin	Infusion, ground, decoction, juice, no preparation	Oral ingestion (eat or drink), wash	Overuse may cause skin irritation	10	0.163
*Phoenix dactylifera* L. (Arecaceae, NA)	Y	F	Nakhel, tamr (،ليخن رمت)	Fruit, seed	Family planning, digestive	Ground, in hot milk or honey	Oral ingestion (drink)	N	1	0.012
*Pimpinella anisum* L. (Apiaceae, EWM_96)	N	S	Yansun (نوسناي)	Seed	Digestive, gynaecological, respiratory, general and unspecified, urological, neurological	Decoction, infusion	Oral ingestion (drink)	Overdose may cause abdominal distension	21	0.559
*Piper nigrum* L. (Piperaceae, EWM_20)	N	S	Flfl aswad, flfl abyad (،دوسا لفلف, ضيبا لفلف)	Fruit	Respiratory, gynaecological, digestive, urological	Ground	Oral ingestion (eat or drink)	Overdose may cause heartburn	6	0.084
*Pistacia lentiscus* L. (Anacardiaceae, EWM_57)	N	–	Mustaka (اكتسم)	Resin	General and unspecified, musculoskeletal	In small pieces on a gauze with lemon	Poultice	N	1	0.008
*Prunus mahaleb L. (Rosaceae, EWM_55)	N	F	Mhallab (بلحم)	Seed	Digestive, general and unspecified	Ground, infusion	Rubbed on gums, topic, oral ingestion (drink), poultice	N	4	0.069
*Psidium guajava L. (Myrtaceae, EWM_94)	N	F	Waraq jawwafa (قرو ةفاوج)	Leaf	General and unspecified, respiratory, neurological	Decoction, infusion	Oral ingestion (drink)	N	7	0.106
*Punica granatum* L. (Lythraceae, EWM_72)	N	F	Roman (نامر)	Peel	Digestive, skin, gynaecological	Ground, decoction	Oral ingestion (eat), poultice, topic	N	7	0.103
*Rhazya stricta* Decne. (Apocynaceae, EWM_30)	Y	–	Harmal (لمرح)	Leaf, root	General and unspecified, digestive	Decoction, ground, infusion	Oral ingestion (drink)	Can cause dizziness and sleeping problems, overdose can be toxic	4	0.08
*Rheum sp. (Polygonaceae, EWM_69)	Y	–	Raoud (دنوار)	Leaf, root	Digestive	Infusion	Oral ingestion (drink)	Not to be used by pregnant or breastfeeding women, or people with kidney problems	1	0.017
*Ricinus communis* L. (Euphorbiaceae, EWM_45)	Y	–	Khurue (عورخ)	Seeds, oil	Digestive, musculoskeletal, skin	Liniment, in juice	Oral ingestion (drink)	Not to be used by pregnant women	2	0.033
*Rosmarinus officinalis L. (Lamiaceae, EWM_03)	N	–	Aklel aljabal (ليلكا لبجلا)	Leaf	Respiratory, general and unspecified, digestive, neurological, cardiovascular	Ground, infusion	Oral ingestion (eat or drink), decoction (steam inhalation)	Not to be used by pregnant women or patients with blood pressure problems	6	0.1
*Ruta chalepensis* L. (Rutaceae, EWM_84)	Y	–	Shathab (باذش)	Leaf	Neurological, ear, respiratory	Decoction	Oral ingestion (drink)	Overdose may cause sleepiness, cirrhosis, abortion	1	0.024
*Salix mucronata Thunb. (Salicaceae, EWM_76)	Y	–	Safsaf (فاصفص)	Leaf	Musculoskeletal	Infusion	Oral ingestion (drink)	N	1	0.003
*Salvia officinalis L. (lamiaceae, EWM_56)	N	–	Miramia (ةيماريم)	Leaf	General and unspecified, digestive, gynaecological, neurological	Decoction, infusion	Oral ingestion (drink), mouthwash	Not to be used during menstruation, increases production of milk	5	0.106
*Senna alexandrina* Mill. (Fabaceae, EWM_78)	Y	–	Sana maki (يكم انس)	Leaf	Digestive, gynaecological	Decoction	Oral ingestion (drink)	Dry, not to be used by pregnant and breastfeeding women	8	0.195
*Sesamum indicum* L. (Pedaliaceae, EWM_89)	Y	F	Smsm (مسمس)	Seed	Neurological, endocrine and nutritional	In food, ground, no preparation	Oral ingestion (eat)	N	2	0.039
*Solenostemma argel (Delile) Hayne (Apocynaceae, EWM_29)	N	–	Hargal (لجرح)	Leaf	Musculoskeletal, digestive, blood and immune system, endocrine and nutritional	Ground, in water	Topic, oral ingestion (drink)	Not to be used by pregnant or breastfeeding women	2	0.042
*Spinacia oleracea L. (Amaranthaceae, EWM_80)	N	F	Sbanekh (خنابس)	Leaf	Blood and immune system, digestive, general and unspecified	With food	Oral ingestion (eat)	N	4	0.052
*Syzygium aromaticum* (L.) Merr. & Perry (Myrtaceae, EWM_66)	N	S	Qrnfol (لفنورق)	Flower bud	Digestive, general and unspecified, endocrine and nutritional, neurological, blood and immune system	Ground, infusion	Oral ingestion (drink), put directly on teeth	Overdose may upset the stomach, not to be used by people with liver problems	14	0.22
*Tamarindus indica* L. (Fabaceae, NA)	Y	F	Tamrr hindi, homar (رمت رمح ،يدنه)	Fruit	General and unspecified, digestive, skin	In water, decoction	Oral ingestion (drink), poultice	N	2	0.034
*Thymus vulgaris* L. (Lamiaceae, EWM_98)	N	–	Zaetir (رتعز)	Leaf	General and unspecified, respiratory, digestive, gynaecological, cardiovascular, neurological	In water or tea, decoction, infusion, in food	Oral ingestion (eat or drink)	Overdose may cause digestion	7	0.114
*Trachyspermum ammi* (L.) Sprague (Apiaceae, EWM_58)	Y	S	Nankha (ةخنان)	Seed	Digestive, musculoskeletal, gynaecological	Ground	Oral ingestion (eat or drink)	Overdose may cause constipation	7	0.062
*Trigonella foenum-graecum* L. (Fabaceae, EWM_31)	Y	S	Helba (ةبلح)	Seed	Family planning, digestive, gynaecological, musculoskeletal, blood and immune system, endocrine and nutritional, urological, respiratory, general and unspecified	Ground in milk, decoction, infusion	Oral ingestion (drink)	Use is not recommended for children under 2, overdose may cause nausea or overweight	24	0.56
*Triticum aestivum L. (Poaceae, EWM_32)	N	F	Janen alqmah (نينج حمقلا)	Seed	Neurological, cardiovascular	Ground	Oral ingestion (eat)	Overdose may cause overweight	1	0.022
*Vigna radiata (L.) R. Wilczek (Fabaceae, EWM_54)	N	F	Mash (شام)	Seed	Neurological, general and unspecified, blood and immune system, musculoskeletal, endocrine and nutritional	With food, ground, decoction	Oral ingestion (eat or drink)	Can cause overweight	8	0.1
*Viola sp. (Violaceae, EWM_08)	Y	–	Banafsj (جسفنب)	Leaf, flower	Respiratory	No preparation	Sublingual, chewing	N	1	0.031
*Vitis* sp. (Vitaceae, EWM_97)	N	F	Zabib (بيبز)	Fruit	Blood and immune system, neurological, endocrine and nutritional	In food, with juice	Oral ingestion (eat or drink)	N	5	0.086
*Zingiber officinale* Roscoe (Zingiberaceae, EWM_100)	N	S	Zanajabil (ليبجنز)	Root	General and unspecified, musculoskeletal, digestive, endocrine and nutritional, cardiovascular, respiratory, neurological, blood and immune system	Decoction, infusion, ground	Oral ingestion (drink)	Overdose may cause heartburn or skin reactions	18	0.294
*Ziziphus spina-christi* (L.) Desf. (Rhamnaceae, EWM_75)	Y	–	Sader (ردس)	Leaf	Skin	Infusion, ground	Wash	N	2	0.015
Not identified (Cupressaceae, EWM_65)	–	–	Qatiran (نارطق)	Oil	Skin	Liniment	Topic	N	1	0.005
Not identified (EWM_79)	–	–	Saq alhamam, khawa jawa (قاس ،مامحلا اوجاوخ)	Root	Skin	Ground	Poultice	N	2	0.049
Not identified (EWM_48)	–	–	Krela (لايرك)	Fruit	Endocrine and nutritional	Ground	Oral ingestion (eat)	N	1	0.031

The modes of preparation and administration for each plant can be found in Table 2. The most used plant parts are leaves (35%), fruits (21%) and seeds (18%). Underground parts (9%), flowers (8%), resin (5%), oil (2%), the whole plant (2%) and bark (1%) are also used. Infusion is the most used mode of preparation (26%), followed by decoction (22%), grinding (22%), mixing with food (7%) and maceration (6%). Plants are sometimes used as they were sourced without any (further) preparation process (5%), juiced (4%) or mixed with dates (2%), milk (1%), honey (1%) or fruits (1%). The most popular modes of administration are oral ingestion as a drink (63%) and eaten (21%). Plants are also administered as poultices (7%) or applied directly on the teeth (2%). They are rarely (1%) used in mouthwash, fumigation and lotions, inhaled, chewed, rubbed or used as washes.

A total of 67 mixtures were documented including combinations of two to four plant ingredients (Table 3). Forty-four medicinal plants (40%) were sometimes used in the mixtures. The medicinal plants most commonly used in mixtures are *Ziziphus spina-christi* (*zanajabil*) cited in 17 mixtures and *Pimpinella anisum* (*yansun*), *Mentha* sp. (*nena*) and *Citrus* sp. (*limon*) each cited in 10 mixtures. The most common mixtures are the combination of *Pimpinella anisum*, *Carum carvi* and *Foeniculum vulgare* used as a digestive, for gynaecological and urological problems; *Pimpinella anisum*, *Cuminum cyminum* and *Foeniculum vulgare* for digestive problems; and *Trigonella foenum-graecum*, *Foeniculum vulgare* and *Pimpinella anisum* for digestive, respiratory and neurological problems. Modes of administration for mixtures are limited to decoctions and infusions and dried and ground plants added to food.

**Table 3:** Mixtures and combinations used by women in Mecca

Mixture	Medicinal plants included (vernacular and scientific names)
Mix1	Qashr albun (*Coffea arabica*), nakhel, tamr (*Phoenix dactylifera*), kamun (*Cuminum cyminum*)
Mix2	Zeetoun (*Olea europaea*), limon (*Citrus* sp.)
Mix3	Zeetoun (*Olea europaea*), dakhun (*Pennisetum glaucum*)
Mix4	Yansun (*Pimpinella anisum*), karawia (*Carum carvi*), shamr (*Foeniculum vulgare*)
Mix5	Marr (*Commiphora myrrha*), rashad, thafa (*Lepidium sativum*), haba sawda (*Nigella sativa*)
Mix6	Shay, shay akhdar (*Camellia sinensis*), kamun (*Cuminum cyminum*)
Mix7	Yansun (*Pimpinella anisum*), kamun (*Cuminum cyminum*), shamr (*Foeniculum vulgare*)
Mix8	Nena (*Mentha* sp.), shay, shay akhdar (*Camellia sinensis*), zanajabil (*Zingiber officinale*)
Mix9	Karawia (*Carum carvi*), helba (*Trigonella foenum-graecum*), kamun (*Cuminum cyminum*)
Mix10	Nena (*Mentha* sp.), shay, shay akhdar (*Camellia sinensis*)
Mix11	Qrnfol (*Syzygium aromaticum*), hal (*Elettaria cardamomum*), qashr albun (*Coffea arabica*)
Mix12	Yansun (*Pimpinella anisum*), shamr (*Foeniculum vulgare*)
Mix13	Helba (*Trigonella foenum-graecum*), shamr (*Foeniculum vulgare*), yansun (*Pimpinella anisum*)
Mix14	Qashr albun (*Coffea arabica*), shamr (*Foeniculum vulgare*), yansun (*Pimpinella anisum*), haba sawda (*Nigella sativa*)
Mix15	Nena (*Mentha* sp.), limon (*Citrus* sp.), zanajabil (*Zingiber officinale*)
Mix16	Limon (*Citrus* sp.), kamun (*Cuminum cyminum*)
Mix17	Yansun (*Pimpinella anisum*), bardaqush (*Origanum majorana*)
Mix18	Marr (*Commiphora myrrha*), karrakum (*Curcuma longa*)
Mix19	Marr (*Commiphora myrrha*), haba sawda (*Nigella sativa*), shamr (*Foeniculum vulgare*), nankha (*Trachyspermum ammi*)
Mix20	Zanajabil (*Zingiber officinale*), shay, shay akhdar (*Camellia sinensis*)
Mix21	Zanajabil (*Zingiber officinale*), qashr albun (*Coffea arabica*)
Mix22	Nankha (*Trachyspermum ammi*), kozbra (*Coriandrum sativum*)
Mix23	Nena (*Mentha* sp.), bardaqush (*Origanum majorana*), habaq (*Ocimum* sp.)
Mix24	Marr (*Commiphora myrrha*), shay, shay akhdar (*Camellia sinensis*)
Mix25	Zanajabil (*Zingiber officinale*), karrakum (*Curcuma longa*)
Mix26	Kaff maryam (Anastatica hierochuntica), halatayta (Ferula assa-foetida), marr (Commiphora myrrha)
Mix27	Basham (*Commiphora gileadensis*), roman (*Punica grantum*), qrnfol (*Syzygium aromaticum*)
Mix28	Nena (*Mentha* sp.), zanajabil (*Zingiber officinale*)
Mix29	Aklel aljabal (*Rosmarinus officinalis*), zanajabil (*Zingiber officinale*)
Mix30	Limon (*Citrus* sp.), zanajabil (*Zingiber officinale*)
Mix31	Limon (*Citrus* sp.), nena (*Mentha* sp.)
Mix32	Qrnfol (*Syzygium aromaticum*), haba sawda (Nigella sativa), zanajabil (*Zingiber officinale*)
Mix33	Haba sawda (*Nigella sativa*), qurfa (*Cinnamomum verum*)
Mix34	Qrnfol (*Syzygium aromaticum*), hal (*Elettaria cardamomum*)
Mix35	Qrnfol (*Syzygium aromaticum*), hana (*Lawsonia inermis*)
Mix36	Basham (*Commiphora gileadensis*), adhkhur (*Cymbopogon schoenanthus*)
Mix37	Zanajabil (*Zingiber officinale*), qurfa (*Cinnamomum verum*)
Mix38	Nena (*Mentha* sp.), limon (Citrus sp.), zanajabil (*Zingiber officinale*)
Mix39	Yansun (*Pimpinella anisum*), shay, shay akhdar (*Camellia sinensis*), nena (*Mentha* sp.)
Mix40	Zanajabil (*Zingiber officinale*), flfl abyad, flfl aswad (*Piper nigrum*)
Mix41	Zanajabil (*Zingiber officinale*), limon (Citrus sp.), qashr albun (*Coffea arabica*)
Mix42	Sana maki (*Cassia senna*), tamrr hindi, homar (*Tamarindus indica*)
Mix43	Nankha (*Trachyspermum ammi*), kamun (*Cuminum cyminum*), shamr (*Foeniculum vulgare*)
Mix44	Kamun (*Cuminum cyminum*), karrakum (*Curcuma longa*)
Mix45	Nankha (*Trachyspermum ammi*), kamun (*Cuminum cyminum*), shamr (*Foeniculum vulgare*), haba sawda (*Nigella sativa*)
Mix46	Bsal (*Allium cepa*), limon (*Citrus* sp.)
Mix47	Helba (*Trigonella foenum-graecum*), yansun (*Pimpinella anisum*), shamr (*Foeniculum vulgare*)
Mix48	Haba sawda (*Nigella sativa*), yansun (*Pimpinella anisum*), qrnfol (*Syzygium aromaticum*)
Mix49	Qashr albun (*Coffea arabica*), zanajabil (*Zingiber officinale*)
Mix50	Hargal (*Solenostemma argel*), raond (*Rheum sp.)*
Mix51	Zanajabil (*Zingiber officinale*), qurfa (*Cinnamomum verum*)
Mix52	Zanajabil (*Zingiber officinale*), nena (*Mentha* sp.), kamun (*Cuminum cyminum*)
Mix53	Nena (Mentha sp.), helba (Trigonella foenum-graecum)
Mix54	Qrnfol (*Syzygium aromaticum*), qurfa (*Cinnamomum verum*), zanajabil (*Zingiber officinale*), hal (*Elettaria cardamomum*)
Mix55	Helba (*Trigonella foenum-graecum*), mash (*Vigna radiata*)
Mix56	Yansun (*Pimpinella anisum*), habaq (*Ocimum* sp.)
Mix57	Qashr albun (*Coffea arabica*), qurfa (*Cinnamomum verum*)
Mix58	Portokal (*Citrus sinensis*), limon (*Citrus* sp.)
Mix59	Mhallab (*Prunus mahaleb*), hana (*Lawsonia inermis*), qrnfol (*Syzygium aromaticum*)
Mix60	Qashr albun (*Coffea arabica*), qurfa (*Cinnamomum verum*), qrnfol (*Syzygium aromaticum*)
Mix61	Nim (*Azadirachta indica*), harmal (*Rhazya stricta*), sader (*Ziziphus spina-christi*)
Mix62	Nankha (*Trachyspermum ammi*), limon (*Citrus* sp.)
Mix63	Nankha (*Trachyspermum ammi*), qashr albun (*Coffea arabica*)
Mix64	Nankha (*Trachyspermum ammi*), haba sawda (*Nigella sativa*), rashad, thafa (*Lepidium sativum*)
Mix65	Rashad, Thafa (*Lepidium sativum*), nakhel, tamr (*Phoenix dactylifera*)
Mix66	Haba sawda (*Nigella sativa*), nakhel, tamr (*Phoenix dactylifera*)
Mix67	Hargal (*Solenostemma argel*), raond (*Rheum* sp.), hndba (chicory), aomloj (*Phyllanthus* cf.)

### Ailments treated with medicinal plants by women in Mecca and remarks on side effects

Medicinal plant uses were documented for 13 etic therapeutic use categories (Table 4). Almost half of the use reports referred to digestive, general and unspecified and respiratory issues, which are common children’s, as well as adult’s, complaints, but do not reflect the most important diseases afflicting the Saudi Arabian population (cardiovascular diseases, diabetes, neuro-psychiatric conditions and injuries [57]). Gynaecological problems, which encompass menstrual cramps and other menstrual disorders, polycystic ovaries, pregnancy and postpartum issues, are fourth in importance both in number of use reports and number of plant taxa used.

**Table 4:** Use reports and number of taxa used for each therapeutic use category

Emic therapeutic use category	Number of use reports	Number of plant taxa
Digestive	215	67
General and unspecified	96	43
Respiratory	70	32
Gynaecological	60	29
Endocrine and nutritional	53	24
Blood and immune system	50	21
Neurological	48	29
Musculoskeletal	38	16
Cardiovascular	36	19
Urological	36	17
Skin	34	16
Family planning	15	9
Ear	2	2

Informants indicated potentially toxic or side effects for almost half of the medicinal plants (*n* = 52, 47%), often associated with inappropriate use (especially overdosing; Table 2). Many observations on plants’ side effects or toxicology made by informants referred to the negative effects of plants on pregnant or breastfeeding women (53% of the plants with noted side effects), or children (11%), and as causing digestive issues (37%) amongst other problems (30%).

### Acquiring medicinal plant knowledge and choosing health care

Social and family networks, as well as mass media, were the two sources of medicinal plant knowledge mentioned by the Meccan women interviewed, who were all responsible for the household health. The same sources have been documented for herbal knowledge among the population of Riyadh [33]. Most elderly women interviewed mentioned that they had learned about medicinal plants from their mothers, grandmothers and neighbours (Table 1). Mecca’s everyday social interactions among women provide plenty of opportunities for the younger generation to learn from older women’s experience about medicinal plant use. Informant 18 (age 60, housewife) mentioned: ‘We always encourage our daughters to help us in the preparation of medicinal plants from early age’. Although some younger women interviewed acknowledged learning about medicinal plant knowledge from their elders, written sources, such as popular books on medicinal plants, were also mentioned as sources of medicinal plant knowledge (Table 1). Women of all ages also mentioned television programs as a source of knowledge about medicinal plants; mass media is put forward by biomedical practitioners as a tool for educational programs to modernise health concepts and make aware of available treatments among the poorly educated [34].

Overall, most of the women interviewed (*n* = 21, 66%) preferred to use medicinal plants rather than biomedicine, but others (*n* = 11, 34%) preferred biomedicine. Medicinal plants were more often preferred by older than younger women (Table 1). However, preference for medicinal plants also varied depending on the ailment that needed treating. Plants were often preferred to treat general malaise, digestive, respiratory, nutritional, neurological, musculoskeletal, cardiovascular, urological, skin and pregnancy-related ailments, some gynaecological problems and anaemia. The women interviewed preferred to use biomedicine in cases of psychological illnesses, eye problems, cancer, unusual gynaecological bleeding, wounds and infectious diseases. Often, they would prefer biomedicine when they suffer an illness for the first time but use medicinal plants for minor, common or chronic ailments. Informant 22 (45 years old) explained: ‘I usually prefer to use medicinal plants to treat my family for diseases that happen continuously such as headache, abdominal pains and menstruation, as well as those that occur due to climate changes such as flu and cough. I use biomedical resources with infectious and psychological diseases, such as depression’. A similar observation was made by Ghazanfar [36] when describing medicinal plant use in the Arabian Peninsula as a whole.

### Medicinal plants used among urban, Muslim women are vastly under-documented

Four books and seven scientific articles published between 1985 and 2014 were retrieved from the literature search (Table 5). Most literature sources on medicinal plants from Saudi Arabia did not provide information on the ethnobotanical methods used nor cite robust plant identification procedures (Table 5), including six studies that did not mention the geographical area in which they were conducted. Of the 11 studies reviewed, plants were identified by experts in nine of them and only four cited one or more botanical keys used to identify collected taxa; eight studies reported the collection of voucher specimens. However, only four of these eight studies reported the voucher numbers in the publications. Four of the studies were identified as rigorous, hence suitable to compare with medicinal plant uses with the list of medicinal plants collected during fieldwork: Ghazanfar [36], Al-Sodany et al. [41], El-Ghazali et al. [58] and Abdulafatih [59] (Table 5). Although studies were selected if there was evidence for accurate plant identification, several plant synonyms were found to be used in these publications (according to The Plant List [54]) and misspelled plant names were not infrequent. Three plant names (8%) used in these literature sources could not be found when cross-checking with The Plant List [54], and six plants cited were identified only at the genus level.

**Table 5:** Reviewed Saudi Arabian medicinal plant literature

Reference	Study area	N plant species identified	Ethnobotanical data collection	Botanical keys used	Botanical identification by experts	Herbaria	Vouchers	Voucher numbers reported
Shalaby et al (1985)—book in English [68]	Not specified	160	Fieldwork to collect plant specimens and records local names	Yes	Yes	Umm Al-Qura University	No	No
Mossa et al (1987)—book in English [35]	Not specified	150	Not reported	No	No	King Saud University	No	No
Abdulafatih (1987)—journal article in English [59]	Asir region and surrounding areas (SW KSA)	61	Fieldwork documentation: local names, plant parts used and medicinal uses, reported	No	Yes	King Saud University (College of Education, Abha Branch)	Yes	Yes
Al-Yaha et al (1990)—book in English [63]	Not specified	150	Not reported	No	Yes	King Saud University	Yes	No
Ghazanfar (1994)—book in English [36]	Arabian peninsula	260	Fieldwork documentation and literature sources (1982–1993)	No	Yes	Sultan Qaboos University (Department of Biology; Sultanate of Oman)	Yes	No
Rahman et al (2004)—journal article in English [69]	Not specified	254	Literature and herbarium sources (College of Pharmacy, King Saud University, National Herbarium of Saudi Arabia)	No	No	King Saud University (College of Pharmacy, King Saud University, National Herbarium of Saudi Arabia)	Yes	No
El-Ghazali et al (2010)—journal article in English [58]	Al-Rass province, Qassim area	47	Fieldwork documentation: interviews with local healers and knowledgeable Bedouin; local names, plant parts used and medicinal uses, reported	No	Yes	Qassim University (Museum of Science, College of Science and Arts, Al-Rass)	Yes	Yes
Al-Sodany et al (2013)—journal article in English [41]	Taif	261 (165 medicinal)	Fieldwork documentation: interviews with local environments. Literature review	Yes	Yes	Kafr El-Sheikh University (Herbarium of Biology Department, Faculty of Science, Taif)	Yes	No
Youssef (2013)—journal article in English [70]	Qassim and central Saudi Arabia	83	Fieldwork documentation: open-ended interviews. Literature review	Yes	Yes	Qassim University	No	No
Shahat et al (2015)—journal article in English [71]	Tanhat protected area, Riyadh	7	Not reported	No	Yes	King Sud University (Medicinal and Aromatic Plants Research Department, National Research Centre; Herbarium of the Faculty of Pharmacy)	Yes	Yes
Yusuf et al (2014)—journal article in English [72]	Not specified	61	Literature review	Yes	Yes	King Saud University (Herbarium of the College of Pharmacy)	Yes	Yes

The four selected literature sources, all documenting medicinal plant knowledge in rural areas, cited 486 different medicinal plant species, compared to the 95 plants we identified at the species level cited by the 32 women interviewed (Table 2). Combining literature and fieldwork sources results in 525 different species used in Saudi Arabia in total. Five plants identified at the genus level were also new citations of these genera used medicinally in Saudi Arabia. Table 6 presents a comparison between Meccan women’s knowledge and each literature source (taking into account only the plants that could be identified at the species level). Medicinal plants known to the Meccan women interviewed contributed 7.4% of the total list: 39 species (41%) cited by women in Mecca were not reported in the selected Saudi Arabian medicinal plant literature (Table 6; indicated by ‘*’ in Table 5), whereas 56 species (59%) had been previously documented. Of the 56 species already cited in the literature, we documented new vernacular names for nine plants (10%). Forty plants were documented in the literature and were cited for different therapeutic applications by the Meccan women interviewed in this study.

**Table 6:** Comparison of medicinal plants used by Meccan women (MW) identified at the species level (*n* = 95) with the inventories from selected literature sources. NA indicates that a source did not provide vernacular names or therapeutic uses

Reference (rural/urban)	Total number of species listed	New citations by MW	Species with different vernacular names	Species with different therapeutic uses
Abdulafatih 1987 (rural) [59]	61	81 (85%)	84 (88%)	85 (89%)
Al-Sodany et al. 2013 (rural) [41]	165	82 (86%)	NA	NA
El-Ghazali et al. 2010 (rural) [58]	47	87 (92%)	88 (93%)	90 (95%)
Ghazanfar 1994 (not specified) [36]	260	43 (45%)	53 (56%)	55 (58%)
Total literature	486	39 (41%)	48 (51%)	79 (83%)

## Discussion

### Edible, traded and Muslim medicinal plants

Urbanisation is often considered an aspect of modernisation that leads to the erosion of medicinal plant knowledge [6], but urban contexts may have vibrant medicinal plant use traditions [3–7]. In the Middle East, urban male herbalists are acclaimed for their specialist medicinal plant knowledge [43]. In this study, we evidence a rich body of female, lay medicinal plant knowledge, supporting the observation by Elolemy and AlBedah [33] that women commonly use herbal therapies in Saudi Arabian cities. Although only 32 Meccan women participated in this study, more than 100 medicinal plants were documented to treat a wide range of health complaints. The knowledge held by women in cities is markedly different from knowledge previously documented, with 41% of the plants cited by Meccan women not in the literature we sourced. This points to the under-documentation of knowledge of medicinal plants in Saudi Arabia but cannot be attributed to male versus female knowledge or urban versus rural knowledge in the absence of further studies. Existing studies may also exclude foods, spices and culinary herbs from ethnobotanical listings of medicinal plants, making less visible knowledge held by women and contributing to the difference we find between published studies and our results.

A third (32%) of the medicinal plants used by the Meccan women interviewed are food plants. Salient food plants cited in this study include onion, celery, cabbage, coriander, lemon, olive oil and dates (Table 2). The use of food plants as medicines by urban populations is widespread [6] and may be due to the easy access to these plants. Medicinal foods are also an important feature of the Mediterranean medical tradition, observed specifically in the Greek Hippocratic texts that influenced Dioscorides’ *Materia Medica* [60], which in turn influenced Arabic medicinal texts [36]. Specific health beliefs associated with foods have also been observed in Arabia [36]. Along with food plants, many medicinal plants reported are spices (17%), which have played a double role as flavouring and medicinal products since the Middle Ages [61]. The most salient medicinal plants identified here are also all spices (*Trigonella foenum-graecum*, *Cuminum cyminum*, *Pimpinella anisum*, *Cinnamomum verum* and *Zingiber officinale*). Spices are both grown in the Middle East and imported from Africa and Southeast Asia (including plants from the Zingiberaceae, Piperaceae, Theaceae, Costaceae, Fucaceae and Musaceae families). This use of imported spices may be a legacy from trade Roman times, when black pepper, ginger, turmeric and cardamom were transported from Southeast Asia into the Mediterranean through Arabian incense trade routes [18, 20]. Moreover, medicinal plant use in urban environments biased towards exotic plants has been observed in Brazil [5] and could also be attributed to easier access to these plants in urban areas. The important use of foods and spices medicinally in cities may be a global characteristic of urban ethnobotanical knowledge, since these are often easily available in urban environments.

Plant availability is a key factor shaping traditional plant use. In urban areas, this does not necessarily reflect the region’s native plant diversity but the plant diversity traded and available in shops and markets. Differences in plant availability between rural and urban contexts may also account for the differences in plant lists reported in this study and published literature. El-Ghazali et al. [58] observed that native plants used by rural populations in Al-Rass province are not traded in domestic markets, and traded plants in shops and markets form the bulk of the medicinal plants available to Meccan women.

Food plants and spices represented half of the medicinal plants used by the Meccan women interviewed (49%), and many of these are plants cited in the Quran or the Hadith. Among Muslims, knowledge of a plant being mentioned in the Quran is often sufficient to validate its medicinal use [62]. Some medicinal plants cited in interviews are mentioned in the ‘the Medicine of the Prophet’: *helba* (*Trigonella foenum-graecum*), *haba sawda* (*Nigella sativa*), *safarjil* (*Cydonia oblonga*), *rashad* (*Lepidium sativum*), *hana* (*Lawsonia inermis*), *zanajabil* (*Zingiber officinale*), *sana* (*Senna alexandrina*), *khull* (*Ammi visnaga*) and *sabr* (*Aloe vera*), according to the list provided by Al-Yahya [63]. Of these, only *safarjil* (*Cydonia oblonga*) had not been documented already in the Saudi Arabian medicinal plant literature, which indicates a widespread influence of the Hadith in traditional Saudi Arabian medicine both in urban and rural environments. Moreover, the common use of mixtures among the research participants matches the recommendation made in ‘the Medicine of the Prophet’ that ‘city dwellers’ require the use of compound drugs (according to Deuraseh [39]). Specific modes of administration recorded among women in Mecca are also recommended by prophetic medicine [37], specifically the use of food, milk, honey and dates as excipients. This further evidences the influence of Islamic medicine in lay medicinal plant use.

### Dynamic female knowledge

As in other Islamic countries, Meccan women are responsible for dealing with most health issues within the household and their medicinal plant knowledge is gender-specific. Gynaecological problems were frequently mentioned and toxicology and remarks about side effects often concerned women’s reproductive or children’s health, which are all references to gender-specific knowledge.

Meccan women may learn about medicinal plants from their family and social networks, but increasingly, written sources and mass media are becoming important sources of knowledge. This, along with a higher preference for biomedical services amongst the younger generation, could result in the erosion of medicinal plant knowledge. Ethnobotanical knowledge erosion has been observed in the Middle East both among herbalists [43] and the general population [36]. The diffusion of non-local knowledge about medicinal plants through mass media is characteristic to urban settings [64] and has a homogenizing effect on oral pharmacopoeias [65]. Mass media often disseminates information on the uses and properties of commercial plants, increasing their visibility [66] and, alongside availability factors, could also contribute to explain the high proportion of food and spices used among the Meccan women interviewed.

Mass media is also used in Saudi Arabia to communicate biomedical education programs [34]. Although these campaigns may be necessary, they favour biomedical knowledge over traditional therapies. Loss of ethnomedicinal and ethnobotanical knowledge may result from different treatment preferences between generations. Higher preference for biomedical treatments among younger Meccan women reflects the same trend among rural Arabic populations elsewhere in Saudi Arabia [36, 43, 58]. Although Press [2] argues that the disregard of faith and the role of family in biomedical diagnosis and treatment are often sufficient to hamper the utilisation of biomedicine, Ghazanfar notes that in the Arabian Peninsula, ‘modern and traditional medicine may be tried [simultaneously], or if one fails the other will be tried – but where modern medicine achieves results, traditional medicine tends to disappear’ [36, p. 1]. Biomedicine in Islamic countries integrates faith and has a religious viewpoint on caring [38], but people may still prefer home remedies for treating minor ailments [30, 36] as observed in this study. Even when biomedicine is growing and herbal remedies may be in decline, medicinal plant use still plays an important role in urban health care [2, 29, 30].

## Conclusion

We join Emery and Hurley [67] in highlighting the vibrant botanical knowledge and practices in urban areas. Women in Mecca are the primary household health carers and hold a singular, lay body of medicinal plant knowledge to treat mostly common ailments. Plant availability in shops and markets, as well as religious texts, seem to play an important role shaping the urban medicinal flora of women in Mecca; we highlight the important medicinal role in urban environments of foods, spices and traded plants in general. Much of this knowledge had not yet been documented, and gender and geographical biases in research may account for the under-representation of urban women’s knowledge in Saudi Arabian medicinal plant literature.

However, medicinal plant knowledge among Meccan women may be eroded and changed with the spread of new knowledge through mass media and preference for biomedical care. Documentation efforts are urgent for the preservation of the diversity of medicinal plant knowledge in the Arabian Peninsula. We propose that scientifically rigorous ethnobotanical and ethnomedicinal research ‘acknowledging the sociocultural heterogeneity within the community being researched’ [32, p. 242] in Islamic settings can be achieved by teams with both female and male ethnobiologists. Al-Sodany et al. [41] have reported that medicinal plants in rural Saudi Arabia are vastly under-documented, but so far, ethnobotanical enquiry of women’s medicinal plants has been even more overlooked.

## Abbreviations

MW: Meccan women
NA: Not available
