# Correction to: Medicinal plants used by women in Mecca: urban, Muslim and gendered knowledge

**DOI:** 10.1186/s13002-017-0197-0

**Published:** 2017-12-15

**Authors:** Afnan Alqethami, Julie A. Hawkins, Irene Teixidor-Toneu

**Affiliations:** 1 Section of Ecology and Evolutionary Biology (EEB), Harborne Building, School of Biological Sciences, University of Reading, Whiteknights, Reading, RG6 6AS UK; 2 Department of Biology, Faculty of Applied Science, Umm Al-Qura University, PO Box 715, Mecca, 21955 Saudi Arabia

## Correction

In the original publication [1] Arabic names in Table 2 were transcribed from left to right. The corrected version of Table 2 can be found as Additional file 1 in this Erratum.

## Additional file

Additional file 1: Comprehensive inventory of the plants listed by women in Mecca including the scientific name and family, whether the plant is found in the Flora of Saudi Arabia and whether it is used as a food or spice, vernacular name(s), part(s) used, therapeutic use categories, preparation, administration, toxicity and side effects, frequency of citation, and Smith’s S. For presence or absence in the Flora of Saudi Arabia, Y = yes, N = no; and for food and/or spice use, F = food and S = spice. Plants not documented in the selected literature are marked with *. (DOCX 53 kb)
