# Supplementary material for: Correction to: Medicinal plants used by women in Mecca: urban, Muslim and gendered knowledge
Source: J Ethnobiol Ethnomed. 2017 Dec 15;13:71. doi: 10.1186/s13002-017-0197-0 (PMC5732485; doi:10.1186/s13002-017-0197-0)
Supplement: Additional file 1: — Comprehensive inventory of the plants listed by women in Mecca including the scientific name and family, whether the plant is found in the Flora of Saudi Arabia and whether it is used as a food or spice, vernacular name(s), part(s) used, therapeutic use categories, preparation, administration, toxicity and side effects, frequency of citation, and Smith’s S. For presence or absence in the Flora of Saudi Arabia, Y = yes, N = no; and for food and/or spice use, F = food and S = spice. Plants not documented in the selected literature are marked with *. (DOCX 53 kb) [file 13002_2017_197_MOESM1_ESM.docx]

**AF 1. Comprehensive inventory of the plants listed by women in Mecca including the scientific name and family, whether the plant is found in the Flora of Saudi Arabia and whether it is used as a food or spice, vernacular name(s), part(s) used, therapeutic use categories, preparation, administration, toxicity and side effects, frequency of citation, and Smith's S.** For presence or absence in the Flora of Saudi Arabia, Y=yes, N=no; and for food and/or spice use, F=food and S=spice. Plants not documented in the selected literature are marked with *.

| **Scientific name (family, voucher)** | **Flora of Saudi Arabia** | **Food (F) or spice (S)** | **Vernacular name(s) (Arabic)** | **Part(s) used** | **Therapeutic use categories** | **Preparation** | **Administration** | **Toxicological remarks and reported side effects** | **Frequency of citation** | **Smith’s S** |
| --- | --- | --- | --- | --- | --- | --- | --- | --- | --- | --- |
| *Acacia nilotica* (L.) Delile. (Fabaceae, EWM_62) | N | - | Qard (قرض) | Fruit, root | Neurological, digestive | Infusion, decoction | Wash, bath | N | 2 | 0.041 |
| *Acacia senegal* (L.) Willd.  (Fabaceae, EWM_77) | N | - | Samg arabi (صمغ عربي) | Resin | Urological, endocrine and nutritional | Infusion | Oral ingestion (drink) | N | 2 | 0.042 |
| *Aerva javanica* (Burm.f.) Juss. ex Schult. (Amaranthaceae, EWM_90) | Y | - | Tarf (طرف) | All plant | Neurological, digestive | Infusion, ground | Oral ingestion (drink), put directly on the teeth | N | 1 | 0.009 |
| * *Alchemilla* sp. (Rosaceae, EWM_68) | Y | - | Rajel alasad (رجل الآسد) | Root | Digestive | Infusion | Oral ingestion (drink) | Not to be used by pregnant women | 1 | 0.01 |
| **Alchemilla xanthochlora* Rothm. (Rosaceae, EWM_01) | N | - | Abat alseda (عبأة السيدة) | Leaf | Gynaecological | Infusion | Oral ingestion (drink) | N | 1 | 0.021 |
| *Allium cepa* L. (Amaryllidaceae, EWM_14) | N | F | Bsal (بصل) | Bulb, tunic | General and unspecified, gynaecological | Juice with lemon, added to food | Oral ingestion (drink or eat), fumigation, placing onions on the bottom of the feet and wear socks | N | 4 | 0.034 |
| *Allium sativum* L. (Amaryllidaceae, NA) | N | F | Thoom (ثوم) | Bulb | General and unspecified, cardiovascular, digestive, ear | Put with food, in water, mash, no preparation | Oral ingestion (eat or drink), put directly on the teeth | N | 7 | 0.069 |
| *Aloe* sp. (Xanthorrhoeaceae, EWM_74) | Y | - | Sabr (صبر) | Leaf flesh | Digestive, gynaecological, skin | Put in water | Oral ingestion (drink), topic on wounds | Causes diarrhoea | 1 | 0.007 |
| *Aloe vera* (L.) Burm.f. (Xanthorrhoeaceae, EWM_73) | N | - | Sabbar (صبار) | Leaf flesh | Skin | No preparation | Topic on wounds or hair | Overdose may cause colon cancer | 2 | 0.041 |
| **Alpinia officinarum* Hance (Zingiberaceae, EWM_43) | N | S | Kholanjan (خولنجان) | Rhizome | Digestive, cardiovascular, skin | Infusion | Oral ingestion (drink) | N | 1 | 0.013 |
| **Ammi visnaga* (L.) Lam. (Apiaceae, EWM_44) | Y | S | Khull (خلة) | Seed, leaf, fruit | Cardiovascular, urological | Infusion | Oral ingestion (drink) | Overdose may cause dryness and hypotension, not to be used by pregnant women | 4 | 0.053 |
| *Anastatica hierochuntica* L. (Brassicaceae, EWM_34) | Y | - | Kaff maryam (كف مريم) | Fruit | Family planning, gynaecological | Infusion | Oral ingestion (drink), topic | Can cause stomach disorders and nausea | 4 | 0.04 |
| *Anethum graveolens* L. (Apiaceae, EWM_81) | Y | S | Shabath (شبث) | Leaf | Digestive | Infusion | Oral ingestion (drink) | Not to be used by those who suffer from kidney disease | 1 | 0.004 |
| **Apium graveolens* L. (Apiaceae, EWM_46) | Y | F | Korfos (كرفس) | Leaf | Gynaecological, digestive, neurological | Infusion, juice, no preparation | Oral ingestion (eat or drink) | Not to be used by pregnant women | 2 | 0.02 |
| **Artemisia judaica* L. (Asteraceae, EWM_87) | Y | - | Shayh (شيح) | All plant | Digestive | Infusion | Oral ingestion (drink) | Not to be used by pregnant women | 1 | 0.007 |
| **Avena sativa* L. (Poaceae, EWM_88) | Y | F | Shuran (شوفان) | Seed | Endocrine and nutritional | Ground | Oral ingestion (eat) | N | 1 | 0.002 |
| *Azadirachta indica* A. Juss. (Meliaceae, EWM_60) | N | - | Nim (نيم) | Leaf | Digestive, general and unspecified | Decoction | Oral ingestion (drink), bath | N | 2 | 0.035 |
| **Beta vulgaris* L. (Amaranthaceae, EWM_09) | Y | F | Banjr (بنجر) | Fruit | Blood and immune mechanisms | Decoction, juice, no preparation | Oral ingestion (drink ore at) | N | 7 | 0.054 |
| *Boswellia sacra* Flueck. (Burseraceae, EWM_50) | N | - | Laban aldakar, laban shahari (لبان الذكر، لبان شحري) | Resin | General and unspecified, respiratory, neurological | Infusion, chewing, fumigation | Oral ingestion (drink) | N | 8 | 0.113 |
| **Brassica oleracea* L. (Brassicaceae, NA) | N | F | Kronb (كرنب) | Leaf | Digestive | Juice (with apples and milk), in food, decoction | Oral ingestion (drink or eat) | N | 7 | 0.018 |
| *Brassica rapa* L. (Brassicaceae, EWM_40) | N | F | Khardal (خردل) | Seed | Musculoskeletal, cardiovascular | Ground, infusion | In hot water (to put feet in) | N | 2 | 0.028 |
| *Calotropis procera* (Ait.) Ait. fil., (Apocynaceae, NA) | Y | - | Eshr (عشار) | Flower | Respiratory | In water, ground | Oral ingestion (drink or eat) | Can cause diarrhoea | 1 | 0.005 |
| **Camellia sinensis* (L.) Kuntze (Theaceae, EWM_85) | N | F | Shay, shay akhdar (شاي, شاي أخضر) | Leaf | Digestive, metabolic and nutritional, gynaecological, general and unspecified, family planning | Decoction, infusion | Topic, oral ingestion (drink), fumigation | When ingested, overdose may cause diarrhoea | 7 | 0.09 |
| *Carthamus tinctorius* L. (Asteraceae, EWM_61) | Y | - | Osforr (عصفر) | Flower | Endocrine and nutritional, cardiovascular | Infusion | Oral ingestion (drink) | Overdose may cause diarrhoea | 1 | 0.03 |
| **Carum carvi* L. (Apiaceae, EWM_37) | N | S | Karawia (كراوية) | Seed | Digestive, gynaecological, general and unspecified, respiratory, family planning | Decoction | Oral ingestion (drink) | Overdose may affect the kidneys | 6 | 0.111 |
| *Ceratonia siliqua* L. (Fabaceae, EWM_41) | N | F | Kharnub (خرنوب) | Fruit | Digestive | Infusion | Oral ingestion (drink) | N | 1 | 0.025 |
| **Cinnamomum verum* J. S. Presl (Lauraceae, EWM_67) | N | S | Qurfa (قرفه) | Bark | Gynaecological, digestive, blood and immune system, endocrine and nutritional, respiratory, family planning | Decoction, ground | Oral ingestion (eat or drink) | Overdose may cause fainting, hypotension, dizziness and sweating, bleeding if used for more than 3 days, not to be used with ginger, not to be used by pregnant women (may cause abortion) | 20 | 0.349 |
| *Citrullus colocynthis* (L.) Schrad. (Cucurbitaceae, EWM_28) | Y | - | Hanzal (حنظل) | Leaf, fruit | Digestive, skin | Infusion, no preparation | Oral ingestion (drink), topic | N | 2 | 0.041 |
| **Citrus sinensis* (L.) Osbeck (Rutaceae, EWM_51) | N | F | Portokal (برتقال) | Fruit | Digestive, general and unspecified | Juice, no preparation | Oral ingestion (eat or drink) | N | 2 | 0.04 |
| *Citrus* sp. (Rutaceae, EWM_51) | N | F | Limon (ليمون) | Fruit | General and unspecified, digestive, endocrine and nutritional, respiratory | Juice, dry until black and ground in water, decoction | Oral ingestion (drink), topic | N | 10 | 0.201 |
| *Coffea arabica* L. (Rubiaceae, EWM_64) | N | F | Qashr album (قشر اللبن) | Pericarp | Blood and immune system, gynaecological, endocrine and nutritional | Decoction, infusion | Oral ingestion (drink) | Not to be used by pregnant women | 6 | 0.117 |
| *Commiphora gileadensis* (L.) C. Christ. (Burseraceae, EWM_12 | Y | - | Basham (بشام) | Root | Gynaecological, general and unspecified, respiratory, neurological | Decoction, infusion | Oral ingestion (drink), topic | N | 3 | 0.051 |
| *Commiphora myrrha* (Nees) Engl. (Burseraceae, EWM_53) | Y | - | Marr (مر) | Resin | Skin, digestive, respiratory, gynaecological, general and unspecified | In water, infusion | Poultice, mouthwash, oral ingestion (drink) | Overdose may upset the stomach and cause general discomfort | 17 | 0.323 |
| *Coriandrum sativum* L. (Apiaceae, EWM_47) | Y | F | Kozbra (كزبرة) | Leaf | Digestive, cardiovascular, neurological | Ground, infusion, with food | Oral ingestion (eat or drink) | Overdose may cause infertility | 5 | 0.079 |
| **Costus* sp. (Costaceae, EWM_63) | N | - | Qasd hindi (قصد هندي) | Root | Endocrine and nutritional, general and unspecified, gynaecological, blood and immune system, urological, family planning | Ground, decoction | Oral ingestion (eat or drink) | N | 4 | 0.05 |
| **Crocus sativus* L. (Iridaceae, EWM_99) | N | S | Zafran (زعفران) | Stigma | General and unspecified, digestive, respiratory | With food, in water | Oral ingestion (eat or drink) | Not to be used by pregnant women | 3 | 0.025 |
| *Cucumis sativus* L. (Cucurbitaceae, NA) | N | F | Khiar (خيار) | Fruit | Urological, digestive, neurological | No preparation | Oral ingestion (eat) | N | 2 | 0.033 |
| *Cuminum cyminum* L. (Apiaceae, EWM_36) | Y | S | Kamun (كمون) | Seed | Digestive, gynaecological, endocrine and nutritional, general and unspecified, respiratory | Infusion, decoction, ground | Oral ingestion (eat or drink) | Overdose may upset the stomach and produce constipation) | 22 | 0.486 |
| *Curcuma longa* L. (Zingiberaceae, EWM_38) | N | S | Karrakum (كركم) | Rhizome | Musculoskeletal, skin, blood and immune system, general and unspecified, digestive, respiratory, endocrine and nutritional | Ground, infusion, with honey | Oral ingestion (eat or drink), poultice | Overdose may upset the stomach | 8 | 0.09 |
| **Cydonia oblonga* Mill. (Rosaceae, NA) | N | F | Safarjil (سفرجل) | Fruit | Digestive | No preparation | Oral ingestion (eat) | N | 1 | 0.02 |
| **Cymbopogon schoenanthus* (L.) Spreng. (Poaceae, EWM_02) | Y | - | Adhkhur (اذخر) | Leaf | Neurological, digestive, urological, general and unspecified | Decoction | Oral ingestion (drink), decoction (steam inhalation) | N | 3 | 0.069 |
| **Dialium* sp. (Fabaceae, EWM_26) | N | - | Hamayd (حميض) | Fruit | Digestive, urological | Infusion | Oral ingestion (drink) | N | 1 | 0.008 |
| **Dipterygium glaucum* Decne. (Cleomaceae, EWM_06) | Y | - | Arfaj (عرفج) | Leaf | Digestive, respiratory | Infusion | Oral ingestion (drink), decoction (steam inhalation) | N | 1 | 0.003 |
| **Dracaena cinnabari* Balf.f. (Asparagaceae, EWM_16) | N | - | Dam alakhwan (دم الأخوين) | Resin | General and unspecified, digestive | Ground | Poultice | N | 1 | 0.006 |
| **Elettaria cardamomum* (L.) Maton (Zingiberaceae, EWM_24) | N | S | Hal (هال) | Fruit, seed | Respiratory | Infusion | Oral ingestion (drink) | N | 1 | 0.004 |
| *Eruca* sp. (Brassicaceae, EWM_33) | Y | - | Jarjir (جرجير) | Leaf | Blood and immune system, respiratory | With food, no preparation | Oral ingestion (eat or drink) | N | 9 | 0.133 |
| *Eucalyptus camaldulensis* Dehnh. (Myrtaceae, EWM_35) | N | - | Kafor (كافور) | Leaf | Neurological, digestive, general and unspecified | Infusion, decoction | Oral ingestion (drink) | N | 3 | 0.035 |
| *Ferula assa-foetida* L. (Apiaceae, EWM_25) | N | - | Halatayta (حلتيتة) | Resin | Digestive, respiratory | Maceration | Oral ingestion (drink) | Not to be used by breastfeeding women, children should not take more than 5ml | 4 | 0.068 |
| *Ficus palmata* Forssk. (Moraceae, NA) | Y | F | Hamat (حماط) | Fruit | Digestive | No preparation | Oral ingestion (eat) | N | 1 | 0.007 |
| *Foeniculum vulgare* Mill. (Apiaceae, EWM_83) | Y | S | Shamr (شمر) | Seed | Digestive, urological, gynaecological, general and unspecified, neurological, blood and immune system | Infusion, decoction, ground | Oral ingestion (drink or eat) | Overdose may cause inflammation of the intestines | 14 | 0.248 |
| **Fucus vesiculosus* L. (Fucaceae, EWM_21) | N | - | Foqus (فوقس) | Leaf | Endocrine and nutritional, cardiovascular | Infusion | Oral ingestion (drink) | N | 1 | 0.004 |
| **Glebionis coronaria* (L.) N.N. Tzvel. (Asteraceae, EWM_04) | N | - | Aqhwan (اقحوان) | Leaf, flower | Digestive | Infusion | Oral ingestion (drink) | Not recommended for babies | 1 | 0.004 |
| *Glycyrrhiza glabra* L. (Fabaceae, EWM_18) | Y | - | Eirqsos (عرقسوس) | Root | Digestive, musculoskeletal | Infusion | Oral ingestion (drink) | N | 1 | 0.023 |
| **Hibiscus sabdariffa* L. (Malvaceae, EWM_49) | N | - | Kwajara karakadi (كوجراتي، كركدية) | Calyx, sepal | Cardiovascular, blood and immune system, digestive, urological, neurological | Infusion, decoction | Oral ingestion (drink) | N | 15 | 0.209 |
| **Hordeum vulgare* L. (Poaceae, EWM_82) | Y | F | Shaeir (شعير) | Seeds | Urological, digestive | Decoction | Oral ingestion (drink) | Not to be used by pregnant women | 5 | 0.041 |
| **Hyphaene* sp. (Arecaceae, EWM_17) | Y | - | Dom (دوم) | Fruit | Cardiovascular | Ground, infusion with honey | Oral ingestion (drink) | N | 1 | 0.026 |
| *Juniperus procera* Hochst. ex Endl. (Cupressaceae, EWM_05) | Y | - | Arar (عرعر) | Fruit | Urological | Infusion, decoction | Oral ingestion (drink) | Not to be used by pregnant women | 1 | 0.006 |
| **Lactuca sativa* L. (Asteraceae, NA) | N | F | Khas (خس) | Leaf | Blood and immune system, urological, digestive | With food | Oral ingestion (eat) | N | 2 | 0.02 |
| **Laurus nobilis* L. (Lauraceae, EWM_93) | N | S | Waraq alghar (ورق الغار) | Leaf | Urological, general and unspecified | Infusion | Oral ingestion (drink) | N | 1 | 0.007 |
| *Lawsonia inermis* L. (Lythraceae, EWM_27) | Y | - | Hana (حنا) | Leaf | Neurological, musculoskeletal, skin | Ground, with honey in water | Topic, poultice | N | 3 | 0.03 |
| *Lepidium sativum* L. (Brassicacea, EWM_70) | Y | S | Rashad, thafa (رشاد، ثفاء) | Seed | Gynaecological, musculoskeletal, skin, digestive, endocrine and nutritional, neurological, general and unspecified, cardiovascular | Ground, infusion, no preparation | Oral ingestion (eat or drink), poultice | Not to be used for over a month, nor by pregnant women, overdose may cause diarrhoea and upset stomach | 13 | 0.228 |
| *Linum usitatissimum* L. (Linaceae, EWM_13) | Y | - | Bidharrat alkitan (بذرة الكتان) | Seed | Digestive, endocrine and nutritional, general and unspecified, gynaecological | Ground, infusion | Oral ingestion (eat or drink) | Can cause stomach and abdominal pain, and gases | 4 | 0.079 |
| **Lupinus albus* L. (Fabaceae, EWM_91) | N | F | Tarmas (ترمس) | Seed | Neurological, endocrine and nutritional, blood and immune system | With food, infusion, decoction | Oral ingestion (eat or drink) | Overdose may cause phlegm and skin yellowing | 4 | 0.035 |
| *Lycopersicon esculentum* Mill. (Solanaceae, NA) | N | F | Tamatum (طماطم) | Fruit | Blood and immune system | No preparation | Oral ingestion (eat) | Overdose may cause diarrhoea | 1 | 0.023 |
| *Malva parviflora* L. (Malvaceae, EWM_39) | Y | - | Khabiza (خبيزة) | Leaf, flower | Family planning, general and unspecified, respiratory | Infusion, no preparation | Chewing, oral ingestion (drink) | N | 1 | 0.002 |
| *Matricaria aurea* (L.) Sch. Bip. (Asteraceae, EWM_07) | Y | - | Babunj (بابونج) | Flower | General and unspecified, neurological, cardiovascular, digestive, respiratory | Infusion | Inhale, oral ingestion (drink) | Overdose may cause dizziness or headache | 9 | 0.134 |
| **Melissa officinalis* L. (Lamiaceae, EWM_52) | N | - | Malisaa (مليسا) | Leaf | Digestive | Infusion | Oral ingestion (drink) | Not to be used by pregnant women or children | 1 | 0.019 |
| *Mentha* sp. (Lamiaceae, EWM_59) | Y | - | Nena (نعناع) | Leaf | Digestive, cardiovascular, general and unspecified, neurological, respiratory, gynaecological | Decoction, infusion | Oral ingestion (drink) | N | 16 | 0.289 |
| *Morus nigra* L. (Moraceae, EWM_95) | N | F | Waraq tawt (ورق توت) | Leaf | Family planning | Decoction | Oral ingestion (drink) | N | 1 | 0.013 |
| **Musa acuminata* Colla (Musaceae, NA) | N | F | Moz (موز) | Fruit | Digestive | No preparation | Oral ingestion (eat) | N | 1 | 0.029 |
| *Myrtus communis* L. (Myrtaceae, EWM_92) | Y | - | Waraq alas (ورق الاس) | Leaf | General and unspecified, respiratory, blood and immune system, cardiovascular | Decoction, infusion | Oral ingestion (drink) | N | 2 | 0.022 |
| *Nigella sativa* L. (Ranunculaceae, EWM_22) | N | S | Haba sawda (حبة سوداء) | Seed | Blood and immune system, general and unspecified, respiratory, gynaecological, musculoskeletal, neurological | Ground, 7 seeds with honey or dates, no preparation | Oral ingestion (eat or drink) | Not to be used by pregnant women, children, or babies, if more than 7 seeds are taken in a day, it can cause abortion, overdose may affect the kidneys | 13 | 0.201 |
| *Ocimum basilicum* L. (Lamiaceae, EWM_71) | N | - | Rihan (ريحان) | Leaf, flower | General and unspecified, respiratory | Infusion | Oral ingestion (drink) | N | 1 | 0.006 |
| *Ocimum* sp. (Lamiaceae, EWM_23) | Y | - | Habaq (حبق) | Leaf | General and unspecified, digestive | Decoction, infusion | Oral ingestion (drink) | Not to be used by pregnant women or children | 3 | 0.04 |
| *Olea europaea* L. (Oleaceae, EWM_101) | Y | F | Zeetoun (زيتون) | Oil, leaf | General and unspecified, endocrine and nutritional, respiratory | Syrup, liniment, infusion, decoction | Oral ingestion (eat or drink), liniment | If boiled (leaves) may become toxic, overdose may cause diarrhoea | 5 | 0.086 |
| **Origanum majorana* L. (Lamiaceae, EWM_11) | N | - | Bardaqush (بردقوش) | Leaf | Endocrine and nutritional, urological, digestive, musculoskeletal, cardiovascular | Infusion, decoction | Oral ingestion (drink) | N | 5 | 0.078 |
| **Pennisetum glaucum* (L.) R.Br. (Poaceae, EWM_15) | Y | F | Dakhun (دخن) | Seed | Digestive | Ground | Oral ingestion (eat) | N | 1 | 0.015 |
| *Petroselinum crispum* (Mill.) Fuss (Apiaceae, EWM_10) | N | F | Baqdunas (بقدونس) | Leaf | Cardiovascular, urological, female, gynaecological, blood and immune system, musculoskeletal, skin | Infusion, ground, decoction, juice, no preparation | Oral ingestion (eat or drink), wash | Overuse may cause skin irritation | 10 | 0.163 |
| *Phoenix dactylifera* L. (Arecaceae, NA) | Y | F | Nakhel, tamr (نخيل، تمر) | Fruit, seed | Family planning, digestive | Ground, in hot milk or honey | Oral ingestion (drink) | N | 1 | 0.012 |
| *Pimpinella anisum* L. (Apiaceae, EWM_96) | N | S | Yansun (يانسون) | Seed | Digestive, gynaecological, repiratory, general and unspecified, urological, neurological | Decoction, infusion | Oral ingestion (drink) | Overdose may cause abdominal distension | 21 | 0.559 |
| *Piper nigrum* L. (Piperaceae, EWM_20) | N | S | Flfl aswad, flfl abyad (فلفل اسود  فلفل ابيض) | Fruit | Respiratory, gynaecological, digestive, urological | Ground | Oral ingestion (eat or drink) | Overdose may cause heartburn | 6 | 0.084 |
| *Pistacia lentiscus* L. (Anacardiaceae, EWM_57) | N | - | Mustaka (مستكا) | Resin | General and unspecified, musculoskeletal | In small pieces on a gauze with lemon | Poultice | N | 1 | 0.008 |
| **Prunus mahaleb* L. (Rosaceae, EWM_55) | N | F | Mhallab (محلب) | Seed | Digestive, general and unspecified | Ground, infusión | Rubbed on gums, topic, oral ingestion (drink), poultice | N | 4 | 0.069 |
| **Psidium guajava* L. (Myrtaceae, EWM_94) | N | F | Waraq jawwafa (ورق جوافة) | Leaf | General and unspecified, respiratory, neurological | Decoction, infusion | Oral ingestion (drink) | N | 7 | 0.106 |
| *Punica granatum* L. (Lythraceae, EWM_72) | N | F | Roman (رمان) | Peel | Digestive, skin, gynaecological | Ground, decoction | Oral ingestion (eat), poultice, topic | N | 7 | 0.103 |
| *Rhazya stricta* Decne. (Apocynaceae, EWM_30) | Y | - | Harmal (حرمل) | Leaf, root | General and unspecified, digestive | Decoction, ground, ifusion | Oral ingestion (drink) | Can cause dizziness and sleeping problems, overdose can be toxic | 4 | 0.08 |
| **Rheum* sp. (Polygonaceae, EWM_69) | Y | - | Raoud (راوند) | Leaf, root | Digestive | Infusion | Oral ingestion (drink) | Not to be used by pregnant or breastfeeding women, or people with kidney problems | 1 | 0.017 |
| *Ricinus communis* L. (Euphorbiaceae, EWM_45) | Y | - | Khurue (خروع) | Seeds, oil | Digestive, musculoskeletal, skin | Liniment, in juice | Oral ingestion (drink) | Not to be used by pregnant women | 2 | 0.033 |
| **Rosmarinus officinalis* L. (Lamiaceae, EWM_03) | N | - | Aklel aljabal (اكليل الجبل) | Leaf | Respiratory, general and unspecified, digestive, neurological, cardiovascular | Ground, infusion | Oral ingestion (eat or drink), decoction (steam inhalation) | Not to be used by pregnant women or patients with blood pressure problems | 6 | 0.1 |
| *Ruta chalepensis* L. (Rutaceae, EWM_84) | Y | - | Shathab (شذاب) | Leaf | Neurological, ear, respiratory | Decoction | Oral ingestion (drink) | Overdose may cause sleepiness, cirrhosis, abortion | 1 | 0.024 |
| **Salix mucronata* Thunb. (Salicaceae, EWM_76) | Y | - | Safsaf (صفصاف) | Leaf | Musculokeletal | Infusion | Oral ingestion (drink) | N | 1 | 0.003 |
| **Salvia officinalis* L. (lamiaceae, EWM_56) | N | - | Miramia (ميرامية) | Leaf | General and unspecified, digetive, gynaecological, neurological | Decoction, infusion | Oral ingestion (drink), mouthwash | Not to be used during menstruation, increases production of milk | 5 | 0.106 |
| *Senna alexandrina* Mill. (Fabaceae, EWM_78) | Y | - | Sana maki (سنا مكي) | Leaf | Digestive, gynaecological | Decoction | Oral ingestion (drink) | Dry, not to be used by pregnant and breastfeeding women | 8 | 0.195 |
| *Sesamum indicum* L. (Pedaliaceae, EWM_89) | Y | F | Smsm (سمسم) | Seed | Neurological, endocrine and nutritional | In food, ground, no preparation | Oral ingestion (eat) | N | 2 | 0.039 |
| **Solenostemma argel* (Delile) Hayne (Apocynaceae, EWM_29) | N | - | Hargal (حرجل) | Leaf | Musculoskeletal, digetive, blood and immune system, endocrine and nutritional | Ground, in water | Topic, oral ingestion (drink) | Not to be used by pregnant or brestfeeding women | 2 | 0.042 |
| **Spinacia oleracea* L. (Amaranthaceae, EWM_80) | N | F | Sbanekh (سبانخ) | Leaf | Blood and immune system, digestive, general and unspecified | With food | Oral ingestion (eat) | N | 4 | 0.052 |
| *Syzygium aromaticum* (L.) Merr. & Perry (Myrtaceae, EWM_66) | N | S | Qrnfol (قرونفل) | Flower bud | Digestive, general and unspecified, endocrine and nutritional, neurological, blood and immune system | Ground, infusion | Oral ingestion (drink), put directly on teeth | Overdose may upset the stomach, not to be used by people with liver problems | 14 | 0.22 |
| *Tamarindus indica* L. (Fabaceae, NA) | Y | F | Tamrr hindi, homar (تمر هندي، حمر) | Fruit | General and unspecified, digestive, skin | In water, decoction | Oral ingestion (drink), poultice | N | 2 | 0.034 |
| *Thymus vulgaris* L. (Lamiaceae, EWM_98) | N | - | Zaetir (زعتر) | Leaf | General and unspecified, respiratory, digestive, gynaecological, cardiovascular, neurological | In water or tea, decoction, infusion, in food | Oral ingestion (eat or drink) | Overdose may cause digestion | 7 | 0.114 |
| *Trachyspermum ammi* (L.) Sprague (Apiaceae, EWM_58) | Y | S | Nankha (نانخة) | Seed | Digestive, musculoskeletal, gynaecological | Ground | Oral ingestion (eat or drink) | Overdose may cause constipation | 7 | 0.062 |
| *Trigonella foenum-graecum* L. (Fabaceae, EWM_31) | Y | S | Helba (حلبة) | Seed | Family planning, digestive, gynaecological, musculoskeletal, blood and immune system, endocrine and nutritional, urological, respiratory, general and unspecified | Ground in milk, decoction, infusion | Oral ingestion (drink) | Use is not recommended for children under two, overdose may cause nausea or overweight | 24 | 0.56 |
| **Triticum aestivum* L. (Poaceae, EWM_32) | N | F | Janen alqmah (جنين القمح) | Seed | Neurological, cardiovascular | Ground | Oral ingestion (eat) | Overdose may cause overweight | 1 | 0.022 |
| **Vigna radiata* (L.) R. Wilczek (Fabaceae, EWM_54) | N | F | Mash (ماش) | Seed | Neurological, general and unspecified, blood and immune system, musculoskeletal, endocrine and nutritional | With food, ground, decoction | Oral ingestion (eat or drink) | Can cause overweight | 8 | 0.1 |
| **Viola* sp. (Violaceae, EWM_08) | Y | - | Banafsj (بنفسج) | Leaf, flower | Respiratory | No preparation | Sublingual, chewing | N | 1 | 0.031 |
| *Vitis* sp. (Vitaceae, EWM_97) | N | F | Zabib (زبيب) | Fruit | Blood and immune system, neurological, endocrine and nutritional | In food, with juice | Oral ingestion (eat or drink) | N | 5 | 0.086 |
| *Zingiber officinale* Roscoe (Zingiberaceae, EWM_100) | N | S | Zanajabil (زنجبيل) | Root | General and unspecified, musculoskeletal, digestive, endocrine and nutritional, cardiovascular, respiratory, neurological, blood and immune system | Decoction, infusion, ground | Oral ingestion (drink) | Overdose may cause heartburn or skin reactions | 18 | 0.294 |
| *Ziziphus spina-christi* (L.) Desf. (Rhamnaceae, EWM_75) | Y | - | Sader (سدر) | Leaf | Skin | Infusion, ground | Wash | N | 2 | 0.015 |
| Not identified (Cupressaceae, EWM_65) | - | - | Qatiran (قطران) | Oil | Skin | Liniment | Topic | N | 1 | 0.005 |
| Not identified (EWM_79) | - | - | Saq alhamam, khawa jawa (ساق الحمام، خواجوا) | Root | Skin | Gound | Poultice | N | 2 | 0.049 |
| Not identified (EWM_48) | - | - | Krela (كريلا) | Fruit | Endocrine and nutritional | Ground | Oral ingestion (eat) | N | 1 | 0.031 |
